# Overarching control of autophagy and DNA damage response by CHD6 revealed by modeling a rare human pathology

**DOI:** 10.1038/s41467-021-23327-1

**Published:** 2021-05-21

**Authors:** Yulia Kargapolova, Rizwan Rehimi, Hülya Kayserili, Joanna Brühl, Konstantinos Sofiadis, Anne Zirkel, Spiros Palikyras, Athanasia Mizi, Yun Li, Gökhan Yigit, Alexander Hoischen, Stefan Frank, Nicole Russ, Jonathan Trautwein, Bregje van Bon, Christian Gilissen, Magdalena Laugsch, Eduardo Gade Gusmao, Natasa Josipovic, Janine Altmüller, Peter Nürnberg, Gernot Längst, Frank J. Kaiser, Erwan Watrin, Han Brunner, Alvaro Rada-Iglesias, Leo Kurian, Bernd Wollnik, Karim Bouazoune, Argyris Papantonis

**Affiliations:** 1 Center for Molecular Medicine Cologne (CMMC), University of Cologne, Cologne, Germany; 2 Cluster of Excellence Cellular Stress Responses in Age-associated Disorders (CECAD), University of Cologne, Cologne, Germany; 3 Medical Genetics Department, Koç University School of Medicine, Istanbul, Turkey; 4 Institute of Molecular Biology and Tumor Research, Philipps-University Marburg, Marburg, Germany; 5 Institute of Pathology, University Medical Center Göttingen, Göttingen, Germany; 6 Institute of Human Genetics, University Medical Center Göttingen, Göttingen, Germany; 7 Department of Human Genetics, Radboud University Medical Center, Nijmegen, The Netherlands; 8 Institute of Neurophysiology, University of Cologne, Cologne, Germany; 9 Cologne Center for Genomics, University of Cologne, Cologne, Germany; 10 Biochemistry Centre Regensburg (BRC), University of Regensburg, Regensburg, Germany; 11 Institute of Human Genetics, University Hospital Essen, Essen, Germany; 12 Research Institute of Genetics and Development, Faculté de Médecine, Rennes, France; 13 Institute of Biomedicine and Biotechnology of Cantabria (IBBTEC), University of Cantabria, Santander, Spain; 14 Cluster of Excellence Multiscale Bioimaging: from Molecular Machines to Networks of Excitable Cells (MBExC), University of Göttingen, Göttingen, Germany; 15 Present Address: Heart Center, University Hospital Cologne, Cologne, Germany; 16 Present Address: Bayer AG, Wuppertal, Germany; 17 Present Address: Institute of Human Genetics, University of Heidelberg, Heidelberg, Germany

**Keywords:** Autophagy, Epigenomics, Genomics

## Abstract

Members of the chromodomain-helicase-DNA binding (CHD) protein family are chromatin remodelers implicated in human pathologies, with CHD6 being one of its least studied members. We discovered a de novo *CHD6* missense mutation in a patient clinically presenting the rare Hallermann-Streiff syndrome (HSS). We used genome editing to generate isogenic iPSC lines and model HSS in relevant cell types. By combining genomics with functional in vivo and in vitro assays, we show that CHD6 binds a cohort of autophagy and stress response genes across cell types. The HSS mutation affects CHD6 protein folding and impairs its ability to recruit co-remodelers in response to DNA damage or autophagy stimulation. This leads to accumulation of DNA damage burden and senescence-like phenotypes. We therefore uncovered a molecular mechanism explaining HSS onset via chromatin control of autophagic flux and genotoxic stress surveillance.

## Abstract (web summary)

Members of the CHD chromatin remodeler family are implicated in human pathologies, however CHD6 remained poorly studied. Here, the authors show that CHD6 binds to and regulates autophagy and stress response genes across cell types. They identify a clinical mutation that affects its ability to recruit cofactors, leading to impaired autophagy induction and DNA repair.

## Introduction

Modulation of DNA accessibility is central to the regulation of eukaryotic genome functions like transcription, DNA replication, or repair^1–3^. One large class of enzymes, the ATP-dependent chromatin remodeling factors, can alter DNA accessibility by removing or repositioning nucleosomal proteins along chromosomes. In mammals, the chromodomain-helicase-DNA binding (CHD) proteins represent the largest family of remodelers. In humans, all nine of its members are characterized by the presence of tandem N-terminal chromodomains and by a central SNF2-like ATPase module. Additional domains allow for the further classification of these large (>200 kDa) proteins into three subfamilies. Subfamily I includes CHD1 and -2 that carry DNA binding domains absent from subfamily II members CHD3-5; these instead have tandem plant homeodomains that bind histone tails. Subfamily III includes CHD6-9, marked by the presence of Brahma/Kismet (BRK) and SANT-like domains closer to their C-termini^4,5^. SANT domains, initially discovered in co-repressor proteins, were later found in different remodeling complex subunits as modules that may couple histone-tail binding to enzymatic activity^6^. The CHD SANT/SLIDE domains, which resemble Myb DNA‐binding domains, are conserved between CHD and ISWI proteins. Data suggest that the SANT and SLIDE modules interact with DNA as one cooperative unit important for tuning DNA binding and nucleosome spacing^7^.

Mutations in subfamily III members have been causally implicated in autism (CHD7 and -8)^8–10^ and CHARGE syndrome (CHD7)^11–13^. CHD6 is a far less studied member of this subfamily. To date, CHD6 has not been functionally linked to a human pathology, but there exist reports of large translocations encompassing its locus in one Pitt-Hopkins patient^14^, in a single case of mental retardation^15^, and in sporadic acute myeloid leukemia incidences^16^. In the only existing *CHD6* mouse model, where exon 12 (encoding its conserved ATPase domain) is lacking, no obvious phenotype apart from mild ataxia was observed^17^. At the molecular level, CHD6 is ubiquitously-expressed and resides alongside RNA polymerases at nucleoplasmic sites of nascent RNA synthesis as part of supramolecular complexes^18^. Recent reports implicated CHD6 in the repression of viral replication^19^, in the topological organization of the *CFTR* locus^20^, and in chromatin remodeling at sites of oxidative DNA damage^21^.

Human cells continuously face genotoxic stress throughout development, and mechanisms are in place to survey and restore any resulting DNA damage. Weakening of these mechanisms leads to DNA damage accumulation and is now understood to cause premature ageing syndromes (known as segmental progerias)^22^. For example, the well-studied Hutchinson-Gilford and Werner progerias stem from mutations in *LMNA* and *RECQL2*, respectively, which promote genome instability^23,24^. Alongside increased DNA damage burden, deregulated autophagy constitutes another ageing hallmark. Autophagy is a housekeeping catabolic pathway essential for recycling long-lived organelles and misfolded proteins, and is activated by various stimuli, including growth factor withdrawal, nutrient deprivation, infection, oxidative stress, or hypoxia^25^. A link between autophagy induction and the DNA damage response was recently documented^26,27^. Interestingly, mouse models with conditional, tissue-specific knockout of key autophagy regulators present age-associated defects^28^.

Hallermann-Streiff syndrome (HSS; OMIM ID: #234100) is a rare congenital disorder characterized by craniofacial and dental dysmorphisms with a specific facial gestalt, eye malformations, distinctive facial features, hair and skin abnormalities, and short stature. Due to its clinical course and progression, HSS is regarded as a premature ageing disorder^29–31^. With just few cases reported to date, and with virtually all reports being descriptive, there is an apparent need for dissecting the molecular pathways underlying HSS^32^. Here, we report a de novo missense mutation in CHD6 from an HSS patient that motivated us to dissect the function of this remodeler. We modeled HSS by generating isogenic induced pluripotent stem cell (iPSC) lines carrying the *CHD6* mutation. Using these lines in genomics, biochemical and functional studies, we provide molecular insights into HSS etiology. We identify CHD6 as a major housekeeping regulator of stress and autophagy response genes. Its HSS mutation interferes with co-factor recruitment to CHD6 target loci, resulting in compromised autophagy flux, DNA damage accumulation, and the development of senescence-like phenotypes.

## Results

### Identification of a putatively-causative HSS mutation and generation of an iPSC model

**Fig. 1:**
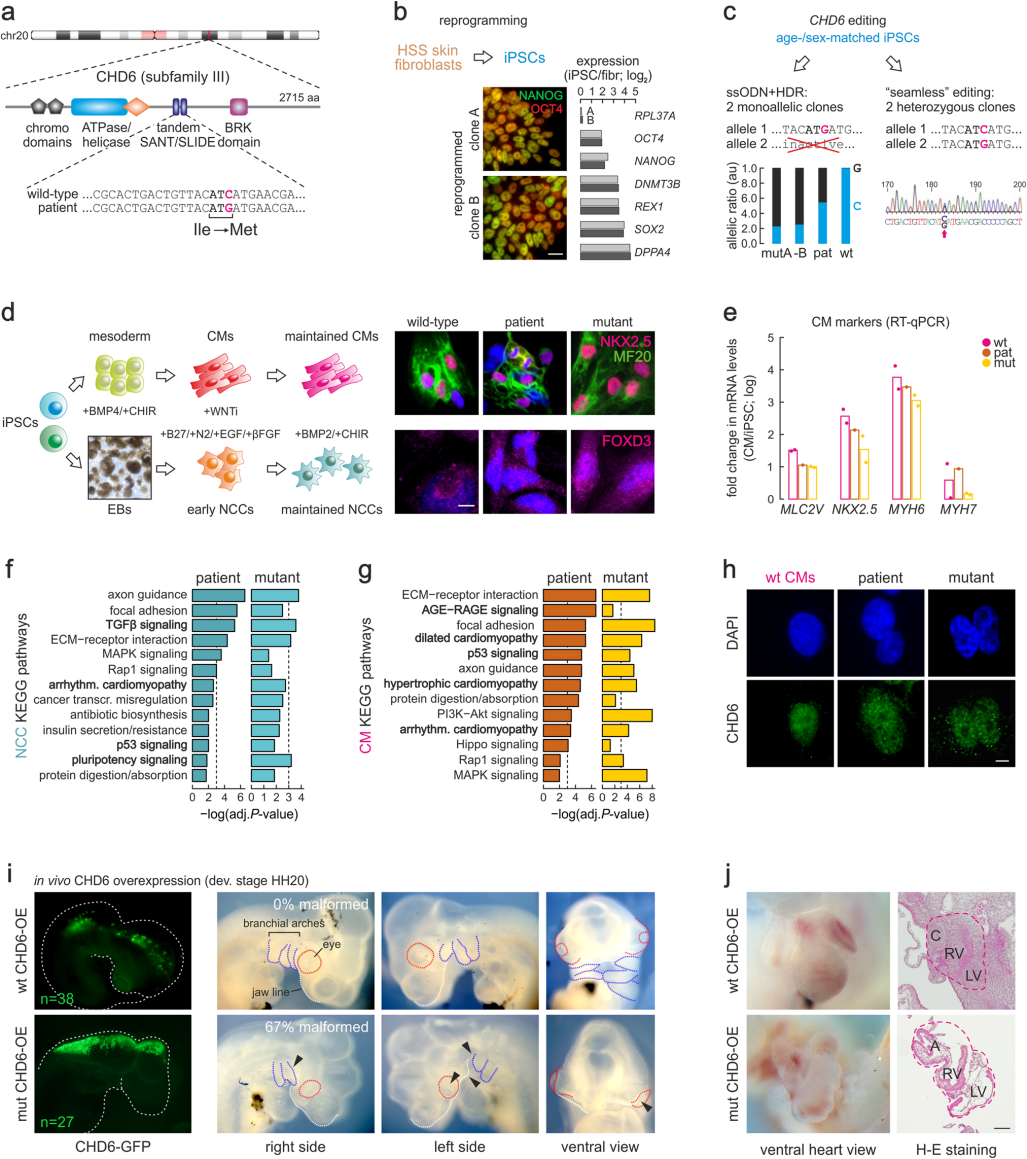
Generation of isogenic iPSCs and developmental impact of the I1600M CHD6 mutation. **a** Schematic representation of the CHD6 protein highlighting key functional protein domains (top) and the HSS-specific I1600M missense mutation in the second SANT/SLIDE domain (bottom). **b** Reprogramming skin fibroblasts from an HSS patient carrying the I1600M CHD6 mutation into iPSCs exemplified by immunofluorescence (left) and pluripotency marker expression levels (mean log_2_ fold-change; right) in two independent clones. Bar, 5 μM. **c** Generation of iPSCs monoallelically (left) or heterozygously expressing I1600M CHD6 (right) using two CRISPR/Cas9 editing strategies. **d** Derivation of wild-type, monoallelic mutant or patient-derived cardiomyocytes (CMs; top) and neural crest cells (NCCs; bottom) from iPSCs and detection of lineage-specific marker genes by immunofluorescence (right). Bar, 5 μM. **e** RT-qPCR mean mRNA level changes of CM markers (*n* = 2 independent replicates, except het) from all genotypes in (**d**). **f** Top KEGG pathways misregulated in both patient and mutant NCCs RNA-seq data. **g** As in (**f**), but using RNA-seq data from patient and mutant CMs. **h** Representative immunofluorescence images of CHD6 distribution in wild-type, patient-derived, and monoallelic mutant CMs. Nuclei were counterstained by DAPI. Bar, 5 μM. **i** Overexpression of wild-type (top row) and mutant CHD6 (bottom row) in chicken embryos and examination of lateral and ventral views at stage HH20 to assess proper development of branchial arches (blue), eyes (red), and jaw (white). The number of embryos analyzed (*n*) is also given. **j** As in (**i**), but for ventral views of the heart (left) and corresponding H-E tissue stainings (right). C conus, RV right ventricle, LV left ventricle, A atrium. Bar, 500 μM.

To understand the molecular basis of HSS, we sought to identify genetic mutations associated with the disease. Despite the rarity of HSS samples, whole-exome sequencing of blood and saliva-derived DNA from a patient and parents uncovered a single de novo missense mutation resulting in an isoleucine to methionine (I1600M) amino acid exchange in the *CHD6* coding sequence. This putatively-causative heterozygous mutation in *CHD6* maps to its predicted second SLIDE domain, at a position which is highly conserved across species and also present in the SLIDE domain of CHD1 (Fig. 1a and Supplementary Fig. 1a). We generated iPSCs from this patient by reprograming fibroblasts from a skin biopsy. Reprograming was confirmed by assessing pluripotency markers at the RNA and protein levels (Fig. 1b and Supplementary Fig. 1b) and by testing the ability of patient-derived iPSCs to form embryonic bodies (Supplementary Fig. 1c) and differentiate in an undirected manner into all three germ layers (Supplementary Fig. 1d–f). As controls for these tests, we used previously characterized age- and sex-matched wild-type iPSCs^33^. However, these lines do not have identical genomic backgrounds, which may complicate data interpretation. To address this, we applied different CRISPR-Cas9 editing approaches to our control iPSCs to generate isogenic lines carrying the *CHD6* mutation. We obtained two independent lines expressing only mutated *CHD6*, and another two lines heterozygously expressing wild-type and mutant *CHD6*, like in the HSS patient (Fig. 1c and Supplementary Fig. 1g). These lines were diploid and karyotypically stable (Supplementary Data File 1), and could be efficiently differentiated into neural crest cells (NCCs) or spontaneously-contracting cardiomyocytes (CMs) (Fig. 1d, e and Supplementary Fig. 1h, i). NCCs were initially chosen because HSS invariably presents as a neurocristopathy^32^, and CMs were chosen since progressing cardiomyopathy has been reported in some patients^34,35^. Collectively, we established a model to study the mechanism underlying HSS in disease-relevant cell types, while also characterizing CHD6 function in both a proliferative and a post-mitotic cellular context.

### The I1600M *CHD6* mutation affects gene expression and development

We first assessed how this *CHD6* mutation affects gene expression by generating transcriptome profiles from all genotypes. Given the importance of NCCs in neurocristopathy-type syndromes, we performed gene set enrichment analysis against the KEGG pathway database using total cell RNA-seq data from two independent clones of patient-derived and gene-edited mutant NCCs. Compared to control cells, we found that focal adhesion, ECM interactions, p53, MAPK, and pluripotency signaling were all affected (Fig. 1f). Interestingly, genes known to be involved in arrhythmogenic cardiomyopathy were also found to be deregulated (Fig. 1f), and we thus turned to CM RNA-seq data analysis. We discovered that many pathways affected in NCCs are also affected in both patient-derived and gene-edited mutant CMs (ECM interactions, p53/MAPK/Rap1 signaling, focal adhesion), while various cardiomyopathy-associated genes were highlighted (Fig. 1g) despite comparable CHD6 levels between the different genotypes (Fig. 1h). Finally, we specifically looked for genes that were commonly deregulated between patient-derived CMs and NCCs, and found >550 genes associated with axon guidance, lipid biosynthesis, and heart contraction to be consistently downregulated compared to wild-type levels (Supplementary Fig. 1j). On the other hand, >450 genes linked to cell adhesion, wound healing, apoptosis, and the MAPK and integrin pathways were consistently upregulated (Supplementary Fig. 1k).

Given that the deregulation of focal adhesion and integrin components are central to cell migration, we hypothesized that, for NCCs especially, the CHD6 mutation could lead to altered migratory potential. However, in vitro migration assays showed no significant differences between wild-type and mutant NCCs (Supplementary Fig. 1l). To also assess this in vivo, we turned to chicken embryos, a well-established model for NCC studies^36,37^. We performed in ovo electroporation to unilaterally overexpress the human wild-type or I1600M mutant CHD6 directly into the neural tube and anterior brain of developing chicken embryos at Hamburger-Hamilton (HH) developmental stage 9 (HH9). Following reincubation, embryo phenotypes were assessed at stage HH20, when wtCHD6-expressing cells displayed the expected migration to the cornea, heart, and along the neural tube (Fig. 1i, top left). In contrast, mutCHD6-expressing cells accumulated more in the neural tube and brain of the embryo (Fig. 1i, bottom left). Critically, 2/3 of embryos displayed severe facial malformations, lack of the 1st, or fusion of the 1st and 2nd branchial arches, enlarged forebrain, as well as diminished mesenchymal tissue, all of which are reminiscent of HSS phenotypes (Fig. 1i). Moreover, we observed strong effects on heart development in mutCHD6-overexpressing embryos. Hematoxylin-eosin (H-E) staining of dissected heart sections showed delayed development of both ventricles, as well as morphological aberrations of the septum (Fig. 1j). These data are in line with our transcriptome analyses and demonstrate how a single CHD6 mutation can severely impact development and gene expression.

### CHD6 binds autophagy-relevant genes across cell types

**Fig. 2:**
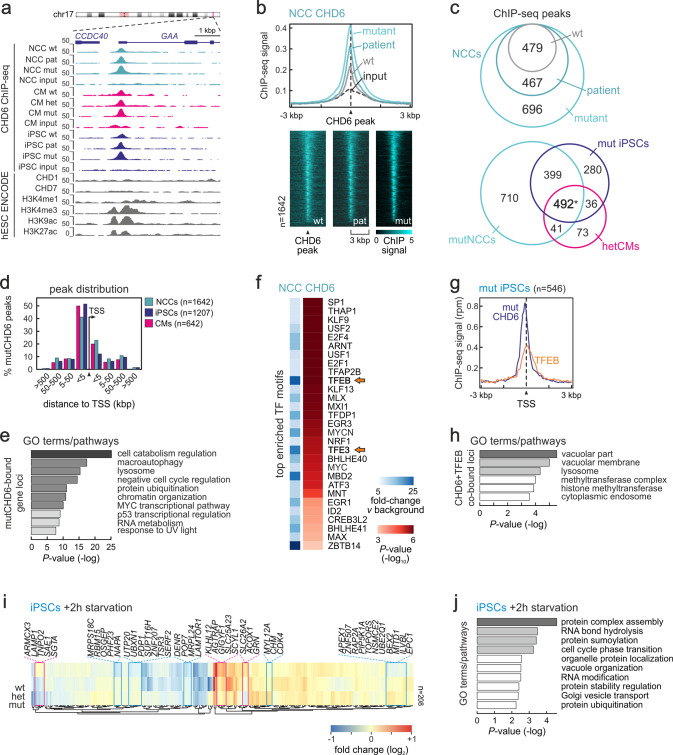
CHD6 binds autophagy-related genes across different cell types. **a** Genome browser views of CHD6 ChIP-seq data from wild-type (wt), monoallelic mutant (mut), heterozygous mutant (het) or patient-derived (pat) NCCs (light blue), CMs (magenta), iPSCs (dark blue) around the *GAA* promoter. hESC ENCODE ChIP-seq data for CHD1/7 and histone marks are aligned below. **b** Line plot (top) and heatmaps (bottom) showing ChIP-seq signal distribution in the 6 kbp around CHD6-bound sites from wild-type (gray), patient-derived (dark blue), or monoallelic mutant NCCs (light blue). Input data provide a baseline (dashed). **c** Venn diagrams showing overlap of CHD6 ChIP-seq peaks from the three indicated NCC genotypes (top) or across cell types (bottom). *: significantly more than expected by chance; *P* < 10^−4^, two-sided hypergeometric test. **d** Bar plot showing the percent of mutCHD6 ChIP-seq peaks located at increasing distances upstream or downstream of gene TSSs from all three cell types. **e** Significantly enriched GO terms associated with mutCHD6-bound genes in NCCs. **f** Heatmaps showing TF motif enrichment (over background; blue shades) and associated *P*-values (red shades) within accessible DNase I footprints overlapping NCC mutCHD6 ChIP-seq peaks. **g** Line plot showing TFEB and mutCHD6 ChIP-seq signal overlap at iPSC peaks. **h** Significantly enriched GO terms associated with the genes at TFEB/CHD6-shared peaks from (**g**). **i** Heatmap showing changes in mRNA levels (log_2_) of mutCHD6-bound and differentially-regulated genes upon 2-h starvation of iPSCs. Those up- or downregulated (magenta/blue rectangles) in both monoallelic- (mut) and heterozygous-mutant iPSCs (het) are highlighted. **j** Significantly enriched GO terms associated with the genes highlighted in (**i**).

To infer which of the deregulated genes above were direct CHD6 targets, we applied a tailored crosslinking and ChIP protocol to iPSCs, NCCs, and CMs of all genotypes (see “Methods”, Fig. 2a and Supplementary Fig. 2a, b). The analysis of our ChIP-seq data collection revealed two unexpected features. First, that mutCHD6, irrespective of the studied cell type, displayed consistently stronger peaks and a larger binding repertoire than wtCHD6 (Fig. 2a–c and Supplementary Fig. 2a–d). Although we cannot formally rule out the possibility that the antibody we used recognizes CHD6 more strongly when it carries the I1600M mutation, our observations possibly signify that mutCHD6 resides longer on chromatin. This was, at least in part, corroborated by ChIP-qPCR enrichment at select gene promoters (Supplementary Fig. 2e) and by cell fractionation western blots in iPSCs (Supplementary Fig. 2f). Second, CHD6 binds many of the same loci across the three cell types, predominantly at gene promoters (Fig. 2c, d and Supplementary Fig. 2a). CHD6-bound loci in NCCs, CMs, and iPSCs were enriched for genes associated with autophagy, lysosome formation, catabolism, cell cycle control, and DNA damage response (Fig. 2e and Supplementary Fig. 2g, h) suggesting that CHD6 may be involved in regulating these processes.

In further support of this, we analyzed accessible transcription factor motifs under CHD6 peaks by combining our ChIP-seq data with publicly-available DNase I footprints from human embryonic stem cells (ENCODE ENCSR143TBZ [https://www.encodeproject.org/annotations/ENCSR143TBZ/]). We found enrichment for TFEB and TFE3 recognition sequences (Fig. 2f). In addition, survey of ENCODE ChIP-seq data returned TFEB as the sole significantly enriched TF signal at CHD6-bound sites (Supplementary Fig. 2i). Given that TFEB is a major autophagy and lysosomal-gene regulator^38^, we performed TFEB ChIP-seq in iPSCs and confirmed it overlaps ~45% of CHD6 peaks (Fig. 2g and Supplementary Fig. 2a). Genes bound by both TFEB and CHD6 were strongly associated with GO terms relevant to autophagy like “lysosome”, “vacuolar membrane” or “cytoplasmic endosome” (Fig. 2h).

Contrary to what was seen in mouse ESCs using endogenously tagged-proteins^39^, CHD6 binding peaks overlapped some CHD2-bound positions, but not those occupied by CHD1 or CHD7, indicating that CHD6 controls disparate pathways (Supplementary Fig. 2j). Thus, we assessed whether CHD6 regulates gene expression in response to autophagy cues and whether the I1600M mutation affects this regulation. To this end, we subjected wt, het-, and mut-iPSCs to 2h of starvation and performed 3′-end RNA-seq. Wild-type iPSCs responded via downregulation of mitochondrial and ribosomal processes, while mutant cells did not. Mut/het-iPSCs also activated ribosome biogenesis and proinflammatory genes, and suppressed those involved in the DNA damage response. Moreover, ERK1/2 signaling, known to regulate subcellular TFEB localization^40^, was also affected (Supplementary Fig. 2k). Looking specifically at CHD6-bound genes, >200 showed levels deviating from wild-type upon starvation (Fig. 2i and Supplementary Fig. 2l). Of these, the genes that showed significant misexpression of the same trend in both het- and mut-iPSCs were linked to processes like RNA hydrolysis and modification, cell cycle transition, vacuole organization, and protein turnover (Fig. 2j). These data again support a ubiquitous role for CHD6 in regulating autophagy genes.

### The CHD6 mutation deregulates autophagy and increases DNA damage burden

To assess the involvement of CHD6 in autophagy regulation, we examined some of its key regulators. First, patient-derived/mutant NCCs displayed elevated phosphorylated ribosomal S6 protein levels (Supplementary Fig. 3a) possibly signifying intensified glycolysis via partial mTORC1 activation. On the other hand, CHD6-mutant CMs displayed decreased phospho-S6 levels, suggesting dampened mTORC1 activity (Supplementary Fig. 3b). However, both hinted towards nuclear depletion of microtubule-associated LC3 protein in mutant compared to wt cells (Supplementary Fig. 3a, b), which implies a tendency to activate autophagy despite the absence of pro-autophagy cues. Both patient-derived and het-CMs show elevated SIRT1 levels compared to wild-type cells (Supplementary Fig. 3c), which could explain LC3 relocalization (from the nucleus to the cytoplasm) due to deacetylation by SIRT1. Again, this occurs in CHD6-mutant cells in the absence of pro-autophagy cues. Together, these results indicate autophagy deregulation in CHD6-mutant NCCs and CMs, while highlighting the differences between the proliferating (NCCs) and post-mitotic context (CMs).

**Fig. 3:**
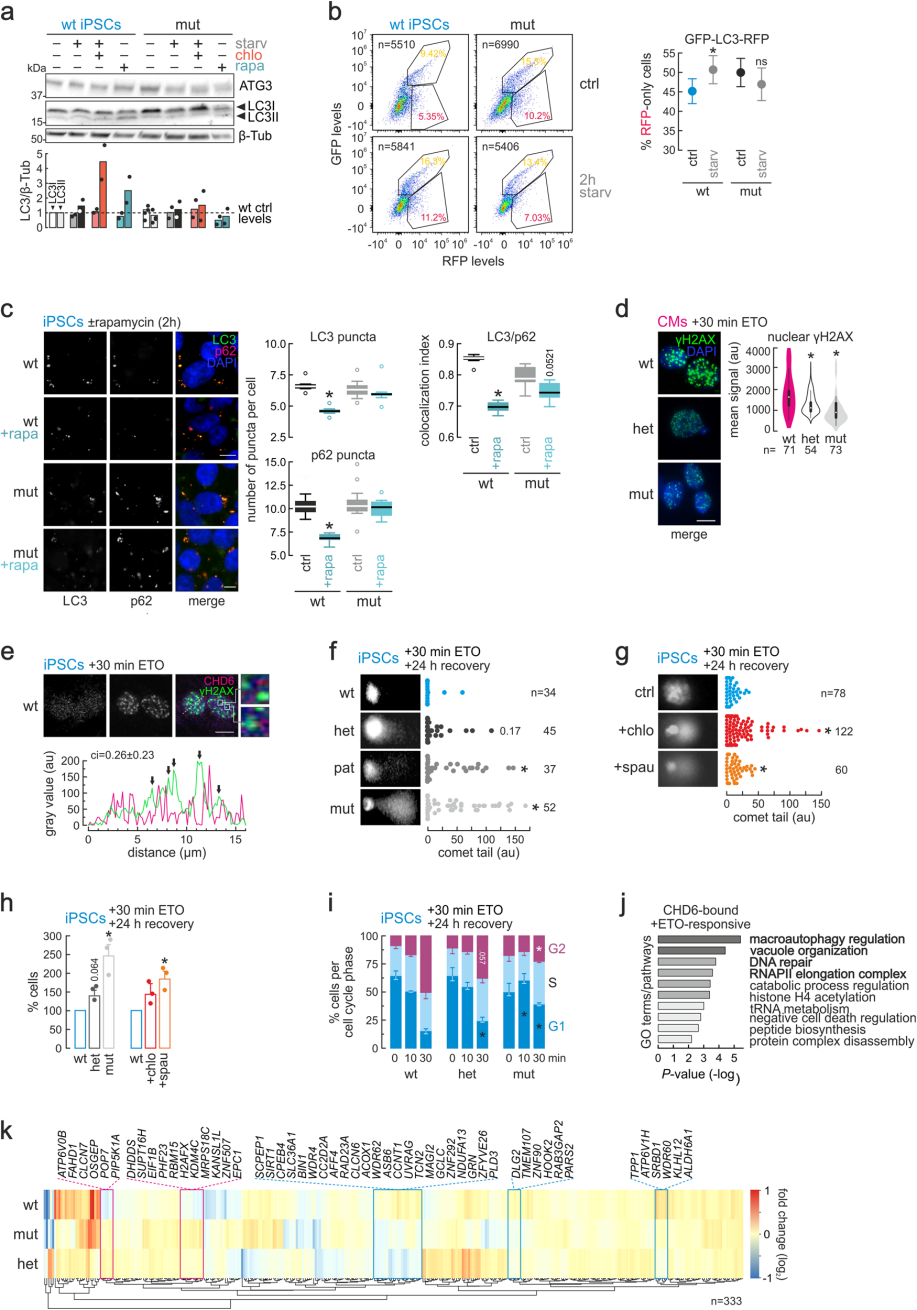
CHD6 affects the DNA damage response through modulation of autophagy flux. **a** Western blots (top) showing changes in ATG3 and LC3 levels in wild-type (wt) and monoallelic mutant iPSCs (mut) that were serum-starved (starv) and/or treated with chloroquine (chlo) or rapamycin (rapa); β-tubulin levels provide a loading control. Mean normalized band intensities from two experiments (±SD) were quantified and plotted relative to wt levels (bottom). **b** FACS profiles (left) of wild-type (wt) or monoallelic mutant iPSCs (mut) transfected with the GFP-LC3-RFP reporter. Plots (right) quantify the percent of RFP-only iPSCs over all RFP/GFP-double positive plus RFP-only cells in control and 2-h starvation conditions (mean ±SD). *: mean significantly different from control; *P* < 0.01, unpaired two-tailed Welsch *t*-test. **c** Representative images of wild-type (wt) or monoallelic mutant iPSCs (mut) immunostained for LC3 (green) and p62 (magenta) in the presence or absence of rapamycin from one experiment are shown. Box plots (mean with whiskers indicating 95th percentile intervals) quantify the number of puncta, mean signal and extent of LC3/p62 colocalization. *: mean significantly different from wt; *P* < 0.01, unpaired two-tailed Welsch *t*-test. Bar, 5 μM. **d** As in (**c**), but for γH2A.X levels (thick black lines indicate IQR, thin lines indicate 95% confidence intervals) after 30-min etoposide (ETO) treatment of wild-type (wt), heterozygous (het), or monoallelic mutant (mut) CMs. *: significantly different from wt; *P* < 0.01, Wilcoxon-Mann-Whitney test. Bar, 10 μM. **e** As in (**d**), but for γH2A.X (green) and CHD6 (magenta) after etoposide treatment of iPSCs. The line scan and colocalization index (ci) of the two signal profiles (bottom; γH2A.X foci indicated by arrows) exemplify the lack of signal overlap. Bar, 5 μM. **f** Comet assays and quantification of tails in wild-type (wt), heterozygous mutant (het), patient-derived (pat) or monoallelic mutant (mut) iPSCs treated with etoposide for 30 min and allowed 24 h to recover; numbers of cells analyzed (n) are indicated. *: significantly different from wt; *P* < 0.05, Wilcoxon-Mann-Whitney test. **g** As in (**f**), but for wild-type iPSCs treated with etoposide and allowed to 24 h recover in the presence or absence of autophagy inhibitors. *: significantly different from wt; *P* < 0.05, Wilcoxon-Mann-Whitney test. **h** Bar plots showing percentage (mean ±SD, *n* = 3 independent experiments) of wild-type (wt), heterozygous (het) or monoallelic mutant (mut) iPSCs that survived apoptosis, also in the presence or absence of autophagy inhibitors. *: mean significantly different from wt; *P* < 0.05, two-tailed unpaired Student’s *t*-test. **i** Bar plots showing the percentage (±SEM, *n* = 3 independent experiments) of wild-type (wt), heterozygous (het) or monoallelic mutant iPSCs (mut) in the G1, S, or G2 cell cycle phase upon 0, 10 or 30 min of etoposide treatment followed by 24 h recovery. *: significantly different from wt; *P* < 0.05, two-tailed unpaired Student’s *t*-test. **j** Significantly-enriched GO terms associated with the genes highlighted in (**k**). **k** Heatmap showing changes in mRNA levels (log_2_) of genes differentially regulated in wild-type iPSCs upon 1 h of etoposide treatment. Those convergently up-/downregulated in monoallelic (mut) and heterozygous mutant (het) cells are highlighted (magenta/blue rectangles).

To further characterize this deregulation, we assessed autophagy flux in iPSCs (used for convenience due to the hypothesized housekeeping nature of autophagy regulation by CHD6). iPSCs with mutant *CHD6* alleles show accumulation of ATG12/ATG5 dimers that are needed for autophagy activation via LC3I conversion to LC3II^38^ (Supplementary Fig. 3d). Upon autophagy inhibition via chloroquine or bafilomycin A1, we observed somewhat higher levels of LC3I isoform in mutant compared to wild-type iPSCs, while upon autophagy induction via short-term (2 h) starvation or rapamycin, LC3II levels in mutant cells remained significantly lower than those in wt iPSCs (Fig. 3a and Supplementary Fig. 3e). In parallel, the levels of the ubiquitin-like conjugating enzyme ATG3 increased upon starvation or rapamycin treatment in wt, but not in mutant iPSCs (Fig. 3a). We then used a GFP-LC3-RFP dual reporter vector^41^ to monitor autophagy induction in living cells (see gating strategy in Supplementary Fig. 4). We found that starvation efficiently induced autophagy and subsequent lysosome fusion in wild-type iPSCs (reflected in the increase in the RFP-positive iPSC fraction), but not in mutant cells (Fig. 3b), which already display significantly fewer lysosomes per cell (Supplementary Fig. 3f). As was observed using resting NCCs and CMs, mutant iPSCs show a tendency to activate autophagy in the absence of pro-autophagy cues. Finally, the levels and number of puncta of p62, another key autophagy regulator, change significantly in response to rapamycin or bafilomycin in wild-type iPSCs, but not in mutant cells where this is dampened (Fig. 3c and Supplementary Fig. 3g). Colocalization between p62 and LC3 is also affected in mutant iPSCs (Fig. 3c), indicative of a reduced capacity to activate autophagy in response to rapamycin. Notably, p62 does not show elevated steady-state levels in *CHD6-*mutant cells (Supplementary Fig. 3g, h). Together, these data implicate CHD6 in the control of autophagy.

Previous studies have shown that autophagy is necessary for efficiently implementing DNA damage responses^26,27^, and we observed that the intrinsic levels and foci of phospho-γH2A.X were significantly reduced across mutant cells (Supplementary Fig. 3d, i, j). Thus, we hypothesized that *CHD6-*mutant cells may suffer from a compromised DNA damage response due to impaired autophagy flux. To test this, we induced DNA double-strand breaks using etoposide and found that γH2A.X foci were weaker and more diffuse in mutant compared to wild-type CMs (Fig. 3d). Interestingly, CHD6 did not redistribute in wild-type cell nuclei in response to etoposide, nor did it colocalize with γH2A.X foci (Fig. 3e). Using iPSC and CM extracts in western blots, we found markedly reduced activation (reflected in phosphorylation) of several DNA damage response components, like p53, CHK1/2, and γH2A.X in mutant cells, as well as inefficient PARP1 cleavage (Supplementary Fig. 3d, k). Moreover, comet assays showed accumulation of unrepaired DNA in iPSCs carrying mutant *CHD6* alleles (Fig. 3f). Such accumulation could be recapitulated in wild-type cells when etoposide treatment was combined with pharmacological inhibition of late-stage autophagy (autolysosome formation and degradation) using chloroquine^42^ or spautin^43^ (Fig. 3g). Impaired DNA damage response also coincided with mutant iPSCs being less susceptible to apoptosis and insensitive to G2/M-phase arrest (Fig. 3h, i), most likely due to impaired signaling (Supplementary Fig. 3d). Notably, this prosurvival effect could be recapitulated in wild-type cells via autophagy inhibition using chloroquine or spautin (Fig. 3h).

Gene expression following DNA damage induction was also affected, as revealed by 3′-end RNA-seq in our iPSC lines. CHD6-bound genes commonly deregulated in both mutant and heterozygous cells were especially enriched for genes associated with macroautophagy, vacuole organization, cell catabolism, RNAP elongation, DNA repair, and cell death (Fig. 3j, k). These expression changes manifested alongside hallmarks of a senescence-like phenotype in mutant iPSCs, most probably due to DNA damage accumulation^44,45^. We recorded reduced levels of H3K27me3-marked heterochromatin (Supplementary Fig. 3l), emergence of HP1α foci in constitutive heterochromatin (Supplementary Fig. 3m), as well as increased β-galactosidase staining (Supplementary Fig. 3n). Importantly, these last two features could be recapitulated following autophagy inhibition by spautin in wild-type cells (Supplementary Fig. 3m, n). Therefore, a functional CHD6 appears to be required for efficient autophagy activation in response to DNA damage.

### CHD6 chromatin binding and remodeling are not affected by the HSS mutation in vitro

The HSS-relevant mutation maps to the CHDCT2 domain of CHD6 (Supplementary Fig. 1a). This domain was initially identified in CHD4 and then in CHD3 and CHD5^46^. Low stringency query of the NCBI Conserved Domain Database (max. *E*-value = 100) uncovered that the CHD6 CHDCT2 is in fact related to the SLIDE (SANT-Like ISWI) domain, which has been implicated in DNA binding^6,7,47^. We, therefore, tested whether the I1600M mutation affected CHD6 binding to DNA or nucleosomes, in vitro, using purified full-length wild-type and mutant CHD6, expressed via a baculovirus system (Supplementary Fig. 5a). mutCHD6 binding was virtually identical to that of wild-type regardless of the template used, as judged by electrophoretic mobility shift assays (EMSAs; Supplementary Fig. 5b). This is consistent with our ChIP-seq data (see Fig. 2a and Supplementary Fig. 2a), and with the fact that the HSS mutation is not at a lysine or arginine residue, both of which were shown to be important for DNA binding by SANT-SLIDE modules^7^. To exclude that additional DNA binding modules in CHD6, like its chromodomains^48,49^, might compensate for the I1600M mutation, we purified and tested the second putative CHD6 SANT-SLIDE module alone in EMSAs. Again, wild-type and mutant CHD6 SANT-SLIDE modules did bind DNA and nucleosomes with similar efficiencies (Supplementary Fig. 5c, d).

We next tested whether the I1600M mutation impinged on the remodeling activity of CHD6. Using restriction enzyme accessibility (REA) assays, we observed that the ability of mutCHD6 to expose RE sites within nucleosomes was indistinguishable from that of the wild-type protein. This held true regardless of whether the RE site was near the nucleosome entry/exit site (*Mfe*I site at +28 bp) or near the pseudo-dyad axis (*Hin*6I at +71 bp; Supplementary Fig. 5e). The ability of CHD6 to expose *Hin*6I sites should require extensive unwrapping or sliding of the nucleosome. This contrasts recent work showing that CHD6, unlike all other subfamily III members, did not slide nucleosomes in vitro, but only disrupted DNA-histone association in a non-sliding manner^50^. Thus, we revisited this aspect using sliding assays. Addition of full-length wtCHD6 to a nucleosome positioned at the end of a 227-bp DNA fragment resulted in the emergence of discrete slower-migrating bands, indicative of histone octamer repositioning in an ATP-dependent manner (Supplementary Fig. 5f). Consistent with REA assays, mutCHD6 activity was indistinguishable from that of the wild-type protein. Finally, we turned to monitoring chromatin remodeling directly at CHD6 target sites. As CHD6 mostly binds to TSSs between the −1 and +1 nucleosomes (Supplementary Fig. 5g), we selected a set of CHD6-bound loci to assess changes in DNA accessibility in iPSCs. We designed primers overlapping nucleosomes directly downstream of CHD6 peaks and used them in MNase-qPCR assays on mononucleosomal templates (Supplementary Fig. 5h, i). All sites analyzed showed weak (~20%) to moderate changes (~40%) in DNA accessibility (Supplementary Fig. 5i), in line with differences inferred from our ChIP-/RNA-seq data. This was also validated genome-wide using ATAC-seq; mutCHD6 peaks exhibited stronger accessibility and their adjacent nucleosome footprint was slightly repositioned (Supplementary Fig. 5j). In summary, the HSS mutation does not appear to affect the enzymatic properties of CHD6 in vitro; however, it does affect chromatin configuration at CHD6 target loci.

### Mutant CHD6 fails to recruit co-factors and activate autophagy genes

**Fig. 4:**
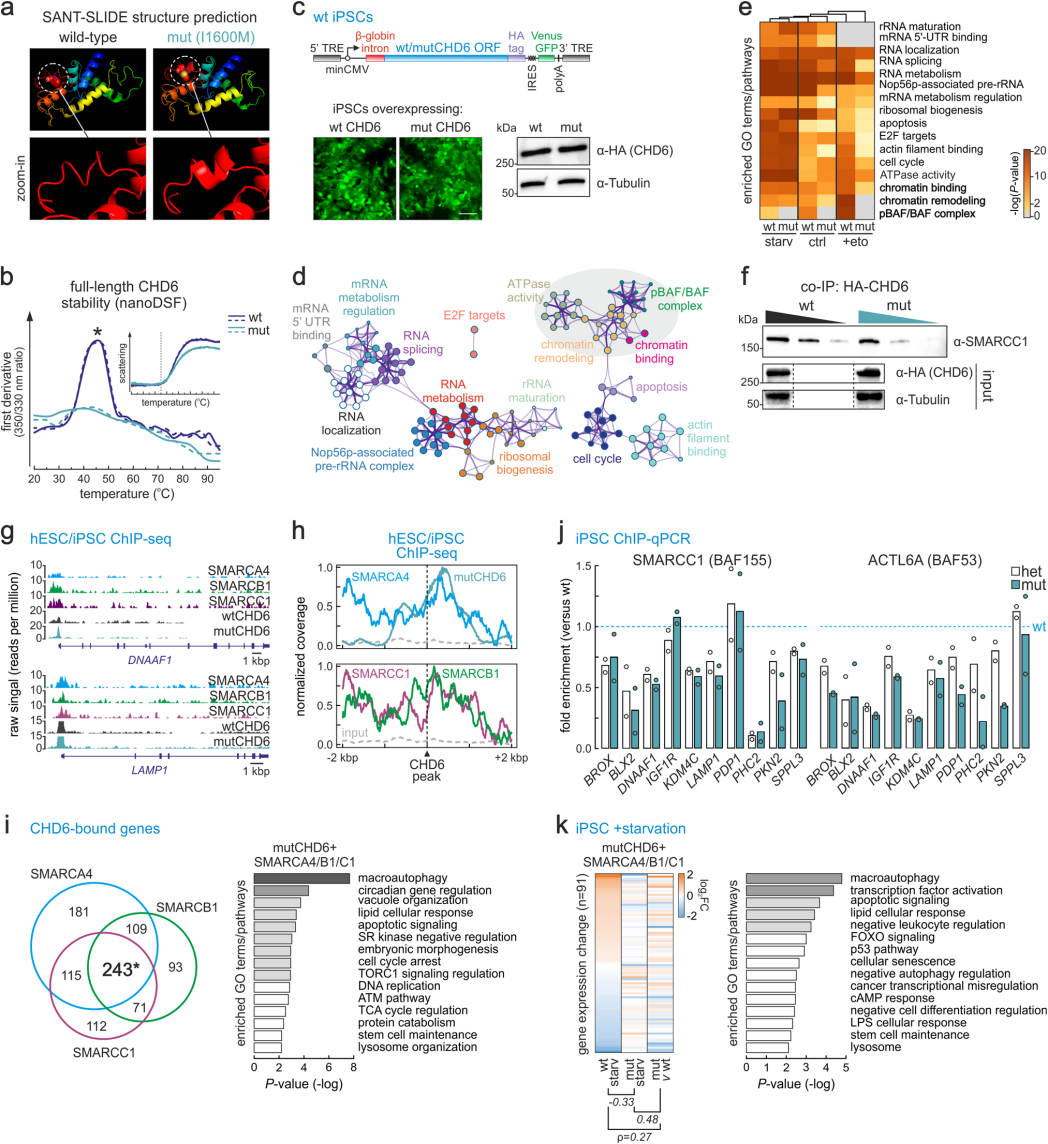
The I1600M CHD6 mutation hinders co-factor recruitment at autophagy gene promoters. **a** In silico rendering of the second putative SANT-SLIDE domain structure for wild-type and mutant CHD6 (based on the published Chd1 SANT-SLIDE structure^7^) and zoomed views around the I1600M mutation (bottom). **b** NanoDSF melting profiles (first derivative of the 350/330 nm ratio; *n* = 3 independent runs) of FLAG-tag purified wild-type (dark blue) or I1600M (light blue) full-length CHD6 along a temperature gradient. *: significantly different mean; *P* < 0.01, two-tailed unpaired Student’s *t*-test. Scattering profiles along the gradient reveal no difference in aggregation (inset). **c** Overexpression of wild-type or mutant *CHD6* cloned into a piggybac vector (left) in iPSCs exemplified by Venus-GFP levels or anti-HA western blotting of CHD6 (right); β-tubulin provides a control. Bar, 25 μM. **d** Network representation of GO terms associated with proteins co-purifying with wild-type CHD6 in steady-state iPSCs. **e** Heatmaps showing enrichment or depletion of GO terms/pathways linked to CHD6-interacting proteins upon starvation, etoposide treatment or in steady-state wild-type and mutant iPSCs. **f** Western blot of SMARCC1 co-immunoprecipitating with overexpressed wild-type (wt) or mutant CHD6 (mut) in iPSCs; anti-HA and anti-β-tubulin blots provide a control. **g** Representative genome browser views for SMARCA4/-B1/-C1 ChIP-seq aligned to CHD6 ChIP-seq from wild-type (wt) and monoallelic mutant iPSCs (mut) around the *DNAAF1* and *LAMP1* promoters. **h** Line plots showing average SMARCA4/-B1/-C1 ChIP-seq signal profiles in the 4 kbp around CHD6 peaks; signal from mutCHD6 ChIP-seq provides a reference. **i** Venn diagram (left) showing overlap of genes bound by mutCHD6 and SMARCA4/-B1/-C1. Bar plots (right) showing significantly-enriched GO terms associated with the 243 shared genes. *: significantly more than expected by chance; *P* < 10^−3^, hypergeometric test. **j** Bar plots showing changes in SMARCC1 or ACTL6A ChIP-qPCR signal (fold enrichment over wt ±SD; *n* = 2 independent experiments) from heterozygous (white) or monoallelic mutant iPSCs (blue). **k** Heatmaps (left) showing changes in mRNA levels (log_2_) in response to iPSC starvation at genes co-bound by CHD6 and SMARCA4/-B1/-C1. Pearson’s correlation coefficients for each pair are shown. Bar plots (right) showing significantly-enriched GO terms associated with these genes.

Our in vitro data suggest that the I1600M mutation does not impede CHD6 association with chromatin or nucleosome sliding. However, we saw strong regulatory effects across all of our HSS-mutant lines. To address this, we first used the I-TASSER^51^, HHpred^52^, and RaptorX^53^ structure prediction tools, and uncovered that the CHD6 region between aa 1448 and 1608 likely folds into a structure highly similar to that of the Chd1 SANT-SLIDE domain^49^ (Fig. 4a, left). Introducing the HSS mutation in this model returned a distorted interface on top of the domain (Fig. 4a, right). Consistent with these predictions, nanoDSF measurements of full-length CHD6 showed that the mutant unfolds at significantly lower temperatures (T*m*: ~41.5 °C) compared to wild-type proteins (~47.7 °C; Fig. 4b), but without differences in aggregation properties (Fig. 4b, inset). Taken together, our data suggest that the I1600M mutation may impact CHD6 folding locally.

Therefore, we asked whether the HSS-mutation interferes with CHD6 protein–protein interactions. We generated iPSC lines stably overexpressing full-length wild-type or mutant HA-tagged CHD6 using a piggybac vector (like in ref. ^45^; Fig. 4c). Following 24 h of induction with doxycycline, we verified that these lines expressed similar levels of wt and mutant CHD6 (Fig. 4c, bottom), prior to co-immunoprecipitating interacting partners. IP eluates were analyzed via quantitative label-free mass-spectrometry. In addition, we also generated CHD6 interactomes upon 2 h of starvation or 1 h of etoposide treatment, and monitored how mutCHD6 interactions compared to wild-type ones (Supplementary Data File 2). In steady-state iPSCs, wtCHD6 presented an interactome consisting of general and specialized transcription factors, other chromatin remodelers, cell cycle regulators, RNA-binding proteins related to RNA processing, and factors involved in rRNA biogenesis (Fig. 4d). When comparing wild-type CHD6 interactors to those of the mutant protein across all three conditions used, the most consistent finding was the depletion of chromatin remodeling complex components, especially those belonging to the BRG-/BRM-associated factor (BAF) and the Polybromo-associated BAF (PBAF) complexes, like SMARCA4 (BRG1), SMARCC1 (BAF155), or ACTL6A (BAF53; Fig. 4e, f and Supplementary Fig. 6a, b). Of note, the loss of interaction between mutCHD6 and SMARCC1 (Fig. 4f) coincided with a loss of interactions amongst BAF/PBAF components, e.g., between SMARCC1 and ACTL6A (Supplementary Fig. 6c). Importantly, this loss was not due to expression changes of the genes involved, as judged by 3′-end RNA-seq data (Supplementary Data File 3).

Both types of canonical BAF and PBAF SWI/SNF complexes in humans are crucial for development and differentiation^54,55^. To address their connection to CHD6-mediated gene regulation, we first used SMARCA4, SMARCB1 (INI1), and SMARCC1 ChIP-seq data from human embryonic stem cells^56^ and found remarkable overlap with CHD6-bound positions (Fig. 4g, h). CHD6 and SMARCB1 ChIP-seq signals were the strongest at their shared sites, compared to all bound positions (Supplementary Fig. 6d). All three BAF/PBAF components share 243 genes with mutCHD6 (and >500 in pairwise combinations; Fig. 4i). These shared genes were linked to processes such as macroautophagy, vacuole organization, apoptosis, cell cycle and DNA replication, or TORC1 signaling (Fig. 4i). ChIP-qPCR at ten of these promoters confirmed reduced recruitment of SMARCC1 and ACTL6A to chromatin in both heterozygous and monoallelic-mutant iPSCs (Fig. 4j), in line with our mass-spec data (Supplementary Fig. 6a).

**Fig. 5:**
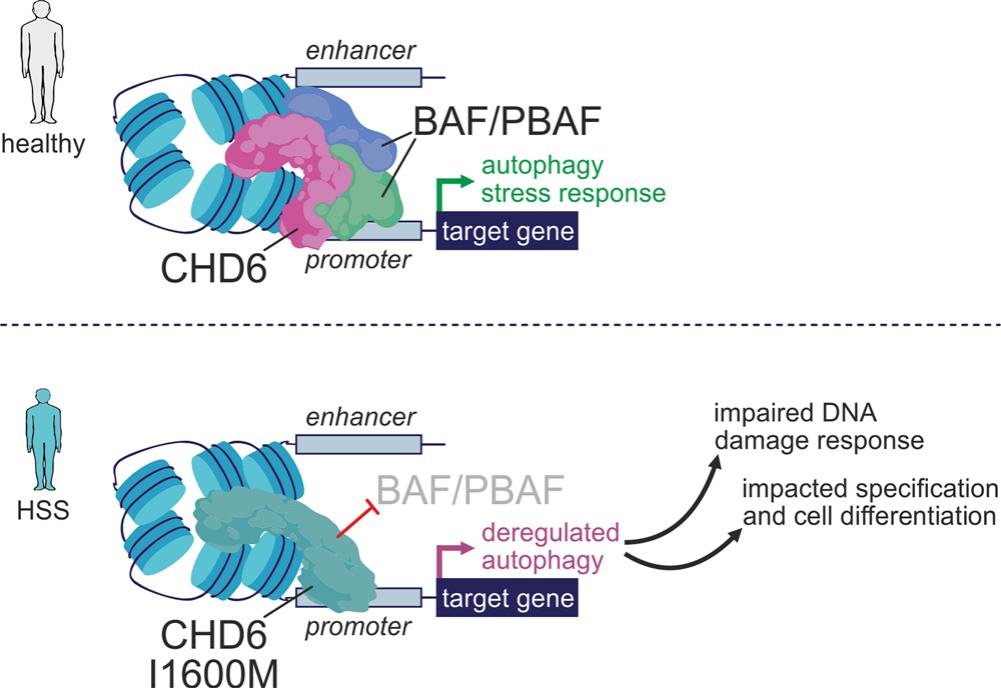
A model for HSS-relevant effects of the CHD6 mutation. In individuals with wild-type CHD6 (top), co-recruitment of CHD6 and BAF/PBAF complexes to the promoters of autophagy genes ensure proper gene regulation in response to pro-autophagy and stress cues. In those carrying the I1600M mutation, the recruitment of BAF/PBAF co-factors is hindered and autophagy control is deregulated; as a result, the DNA damage response is compromised, senescent cell features emerge, and cell differentiation and specification processes can be impacted.

To further investigate the connection between CHD6 and BAF/PBAF, we reanalyzed RNA-seq data from *SMARCB1*-knockdown human embryonic stem cells^57^; 140 *SMARCB1*-regulated genes were also bound by CHD6, >40% of which were downregulated, and associated with lysosomal and vacuole organization, protein stability, chromatin modifiers, and the p53 pathway (Supplementary Fig. 6e, f). Moreover, the expression of ~40% of all BAF/PBAF- and CHD6-cobound genes was significantly altered in mutant compared to wild-type iPSCs in response to autophagy induction via starvation. This strong deviation from the normal transcriptional response concerned genes involved in autophagy, apoptosis, transcription factor activation, the p53 pathway, and cellular senescence (Fig. 4k). Similarly, ~150 genes co-bound by the three BAF/PBAF components and CHD6 also showed misexpression in response to either starvation or etoposide treatment (Supplementary Fig. 6g, h). As regards the latter, DNA damage induction caused significant misregulation of 35% of all BAF/PBAF- and CHD6-bound genes and those genes were linked to MAPK/p53 signaling, DNA replication, cell cycle, and inflammation (Supplementary Fig. 6g). In summary, this data supports the notion that the HSS mutation precludes CHD6 from recruiting additional chromatin remodeling factors to its target loci. This may, in turn, affect proper transcriptional control in response to pro-autophagy or DNA damage cues and lead to dampened autophagy flux and increased DNA damage burden (see model in Fig. 5).

## Discussion

We exploited a CHD6 de novo mutation linked to the rare Hallermann-Streiff syndrome to model the disease using isogenic iPSC lines and investigate the roles of this understudied remodeler. Our work uncovers a potentially important role for CHD6 in autophagy regulation, which could explain its ubiquitous expression across human tissues (The Human Protein Atlas CHD6 [https://www.proteinatlas.org/ENSG00000124177-CHD6/tissue]). In line with this, CHD6 displays remarkable overlap of target loci across very diverse cell types (iPSCs, NCCs, and CMs). A considerable fraction of these loci is co-bound by TFEB, suggesting that they are primed in resting cells for prompt transcriptional response to pro-autophagy stimuli.

The HSS-relevant I1600M CHD6 mutation does not impact its ability to bind or remodel chromatin in vitro. Nevertheless, CHD6-bound genes in mutant cells fail to properly respond to starvation or DNA damage stimuli, and overexpression of mutCHD6 results in severe developmental malformations reminiscent of the HSS phenotype. We can likely attribute this to the inability of the mutant CHD6 putative second SANT-SLIDE domain to interact with and recruit co-remodelers to its target promoters. In our proteomics data, the BAF/PBAF complexes appear as major co-players. The PBAF complex has already been shown to interact with CHD7 to direct NCC formation via enhancer regulation^54^. Interestingly, PBAF was recently also implicated in stress responses^58^ and euchromatic DNA lesion repair^59^. This aligns well with the fact that CHD6 is exclusively found in euchromatin (see ref. ^18^ and own ChIP-seq data), but not at CHD7 sites, hence representing a separate regulatory circuit. Moreover, reanalysis of *SMARCB1*-knockdown deregulated genes in hESCs (SMARCB1 is a BAF/PBAF subunit operating on both promoters and enhancers^55,60^) showed that they overlap CHD6 at lysosomal, autophagy, and DNA damage response genes. This further highlights a requirement for multiple, competing or converging, remodeling activities acting at the same loci to ensure its precise regulation^61^.

In addition, CHD6 might also function through the regulation of post-transcriptional steps; it is worth noting that a considerable subset of CHD6-bound genes is involved in the metabolism and processing of RNA. CHD6 itself interacts with a number of RNA-binding and processing factors in our proteomics data. Interactions with many of these factors are specifically hindered following etoposide treatment of CHD6-mutant cells. These observations possibly further link CHD6 to the control of DNA damage responses and merit future investigation.

In a recent report implicating it in the oxidative DNA damage response, CHD6 was shown to be stabilized via reduced degradation and to relocate rapidly to sites of DNA damage^21^. Like Moore and co-workers, we saw no change in *CHD6* expression upon etoposide-induced DNA damage (or upon starvation), nor did CHD6 redistribute to etoposide-induced DNA lesions. Thus, our data further support that CHD6 is not recruited to DNA double-strand breaks (but only to sites of oxidative damage). Given the potential housekeeping role of CHD6 in autophagy in normal human cells, the accumulation of unrepaired DNA in CHD6-mutant cells possibly results from impaired autophagy. With aberrant autophagy also being a cancer hallmark^62^, the broad mutational spectrum of CHD6 in cancer specimens might therefore contribute to the accumulation of tumor-promoting mutations (COSMIC CHD6 [https://cancer.sanger.ac.uk/cosmic/search?q=CHD6]).

Increased burden of poorly resolved DNA damages is also a hallmark of ageing, and mutations in the DNA damage response machinery give rise to premature ageing-like phenotypes, as seen in ERCC1-^63^ or XPA-deficient cells^64^. We observed acquisition of senescence-like features in CHD6-mutant cells. Interestingly, mutations in some SWI/SNF subunits also accelerate cellular ageing^65^. Our data suggest that CHD6 operates upstream of these changes by coordinating autophagy and DNA damage responses across cell types. In line with this, the key regulator p62 normally mediates the effects of autophagy onto DNA repair mechanisms, likely in conjunction with p53^66^; this is gradually impaired with age, but restored via lifespan-extending interventions^26^. In CHD6-mutant cells, p62 levels are lower and the autophagy-to-DNA damage repair axis disturbed. Thus, we propose that deficient autophagy-driven DNA damage burden and the induction of cellular ageing processes contribute to the onset and progression of HSS.

## Methods

### Whole-exome sequencing (WES) and reprogramming of HSS fibroblasts

Exonic and adjacent intronic sequences were enriched from genomic DNA isolated from blood and saliva of the index patient and the parents using the NimbleGen SeqCap EZ Human Exome Library v2.0 enrichment kit, and sequenced on a HiSeq2000 platform (Illumina). WES data analysis and filtering of mapped targeted sequences was carried out using the “Varbank” exome and genome analysis pipeline v2.6 of the Cologne Center for Genomics (CCG, University of Cologne, Germany); we obtained a mean coverage of 75–95 reads, with 95.9–96.7% of target sequences covered more than 10×. Trio-WES data were filtered for high quality (coverage of >6 reads, minimum quality score of 10), rare autosomal recessive and de novo variants (i.e., with minor allele frequency of <0.5% in the 1000 Genomes database, the Exome Aggregation Consortium browser, and not annotated in all in-house WES datasets of the CCG). Primary fibroblasts from the index patient were isolated via standard skin biopsy, and all downstream work was performed in accordance to the Helsinki Declaration protocols and reviewed and approved by the local institutional Ethics boards (University Hospital Cologne, Germany; University Medical Center Göttingen, Germany).

### iPSC culture and differentiation into cardiomyocytes or neural crest cells

iPSCs were grown in FTDA media^67^. Once confluent, cells were dissociated using accutase at 37 °C for 10 min (Sigma-Aldrich) and 450–600,000 cells were seeded per well of a 6-well plate. Differentiation into cardiomyocytes^68^ used confluent iPSCs that were dissociated into single cells using accutase. Cells were then counted and 600,000 cells for each well of a 24-well plate were aliquoted and spun for 2 min at 300 × *g* at room temperature. The cell pellet was resuspended in ITS medium (knockout DMEM, 1 × x penicillin/streptomycin/glutamine, 1× ITS supplement, 10 μM Y-27632, 25 ng/ml FGF2, 1–2 ng/ml BMP4 and 1–2 μM CHIR99021) and seeded in matrigel-coated 24-well plates. Note that wt-iPSCs required 1.75 μM and 1.25 ng/ml, mut-iPSCs required 1.75 μM and 2 ng/ml, and patient-derived iPSCs required 1.75 μM and 1.75 ng/ml of CHIR and BMP4, respectively, for differentiation. To ensure the equal distribution and attachment of cells, plates were moved crosswise, tapped several times and left for 20 min at room temperature before being transferred into the incubator. After 24 h, ITS growth medium was replaced by TS medium [knockout DMEM, 1x penicillin/streptomycin/glutamine, 1× TS supplement (100× stock: 5.5 μg/ml Transferrin and 6.7 ng/ml Sodium Selenite in 100 ml sterile PBS), and 250 μM 2-phospho-L-ascorbic acid]. After 48 h, TS medium was replenished and supplemented with 10 μM of the Wnt-inhibitor IWP-2 for 48 h. After 48 h, media were changed to fresh TS until beating cells were observed on day 8. At this point, the medium was changed back to knockout DMEM supplemented with 2% FCS, L-glutamine and 1× penicillin/streptomycin for maintenance until cells were used for downstream analysis. Differentiation into neural crest cells^36^ used iPSCs clusters transferred into neural induction media [NIM; 1:1 ratio of DMEM-F12 (Invitrogen #10565-018) and Neurobasal media (Invitrogen #21103-049) complemented with 0.5× N2, 0.5× B27, 5 µg/ml insulin, 20 ng/ml βFGF, 20 ng/ml EGF, 1× pen/strep] in uncoated polypropylene dishes. Cell-spheres/-rosettes were then allowed to spontaneously attach and after 6–9 days hNCLCs (human neural crest-like cells) migrated out of these. Subsequently, rosettes were dissected away and P0-isolated NCLCs were dissociated using accutase and replated for 10–15 days of maintenance in NIM in dishes coated with 1 µg/ml fibronectin.

### Generation of isogenic iPSC lines via CRISPR-Cas9 genome editing

gRNAs were designed against DNA stretches within exon 31 of *CHD6* or its preceding intron using an online tool [http://crispr.mit.edu]. Each gRNA was assembled from two complementary oligonucleotides containing the “NGG” PAM sequence and distinct 4 bp-overhangs (“CACC” and “AAAC”) allowing for cloning into the *Bbs*I restriction site of the pX330A vector^69^. Then, a single-stranded oligonucleotide donor (ssODN)^70^ or a plasmid carrying homology arms^71^ were provided as templates for homologous recombination. iPSCs were seeded at low density in a 6-well plate (175,000 cells/well) and transfected 12 h later by the dropwise addition of a mixture of 200 µl OptiMEM (Invitrogen), 12 µl FuGene HD (Promega) and a total of 3 µg from all plasmids. After 24 h, GFP expression was detectable microscopically; transfection efficiency was estimated to be ≥60%. Cells were then selected in 0.5 µg/ml puromycin for 24 h and reseeded in clonal dilution (5000–8000 cells/well of a 6-well plate). Individual clones were screened using PCR and, ultimately, insertion of the desired mutation was verified by Sanger sequencing. As control lines, two “no gRNA” iPSC clones and two transfected with a gRNA targeting the “safe harbour” AAV locus on chr19 were used interchangeably. All gRNAs/ssODNs used are listed in Supplementary Data File 4.

### CytoScan genome analysis of iPSC lines

Genome integrity of all CRISPR-edited and reprogrammed iPSC lines was assessed using CytoScan HD microarray technology (ThermoFisher Scientific), which allows reliable detection of 25–50 kbp-long copy number changes genome-wide. For this analysis, intact genomic DNA was isolated using the Quick DNA Miniprep kit (Zymo Research) as per the manufacturer’s instructions, and data analyzed via the ChAS suite v4.0 against the reference genome assembly (hg19). A summary is provided in Supplementary Data File 1.

### Generation and analyses of total RNA-seq

Cells from different genotypes/differentiations were harvested in Trizol (Life Technologies) and total RNA was isolated and DNase-treated using the Direct-zol RNA miniprep kit (Zymo Research) as per the manufacturer’s instructions. In the case of cardiomyocytes, cells at day 10–11 of differentiation were used, while in the case of NCCs, cells were obtained by collecting migratory cells from dissected and re-plated rosettes. Barcoded cDNA libraries were generated using the TruSeq RNA library kit (Illumina) via selection on poly(dT) beads. The resulting libraries were paired-end sequenced to >50 million read pairs on a HiSeq4000 platform (Illumina). Transcript quantification was performed using Kallisto v0.44.0^72^, which pseudoaligns RNA-seq reads to transcripts (Ensembl annotation used: GRCh37, comprising all known protein-coding sequences from hg19). After transcript quantification, pseudo-counts were further processed via Sleuth^73^, which bootstraps Kallisto output to ascertain and correct for technical variation. To test for association between gene expression and genotype, we created a linear model and tested the effect of the *CHD6* mutation in each cell type via gene-level analysis. *P*-values were corrected for multiple hypothesis testing using the Benjamini-Hochberg algorithm and an FDR < 0.05 as a significance threshold for all figures and tables. Data preprocessing and visualization were performed using R v3.3.3 and Bioconductor v3.4, and GO term enrichment using Metascape^74^. Differentially-regulated genes per each line are listed in Supplementary Data File 5. For qPCR, the SYBR Green JumpStart Taq ReadyMix (Sigma-Aldrich) was used as per the manufacturer’s instructions.

### 3′-end RNA sequencing and analysis

3′-end RNA-seq was used as a lower-cost alternative to mRNA-seq to interrogate multiple conditions and genotypes. Libraries were prepared from total RNA using the QuantSeq 3′ mRNA-Seq Library Prep Kit (Lexogen), and single-end sequenced on a HiSeq4000 platform (Illumina) generating ~15 × 10^6^ 100 nt-long reads per sample. Reads were quality assessed and mapped to hg19 using STAR^75^. Reads uniquely mapping to exons were quantified using HTSeq-count and differential gene expression was assessed using DESeq2^76^. Differentially-regulated genes per each cell line and treatment are listed in Supplementary Data File 3.

### ChIP-seq and data analysis

For each batch of ChIP experiments, ~12 million cells were crosslinked in 2% PFA for 45 min at 4 °C. From this point onward, cells were processed via the ChIP-IT High Sensitivity kit (Active motif) as per the manufacturer’s instructions, but using the NEXSON protocol for nuclei isolation^77^. Chromatin was sheared to 200–500-bp fragments on a Bioruptor Plus (Diagenode; 2× 20–26 cycles of 30 s “on” and 30 s “off” at the highest power setting), and immunoprecipitations were carried out by adding 4 μg of the appropriate antibodies (CHD6, Bethyl A301-221A; TFEB, Bethyl A303-673A) to ~30 μg of chromatin and incubating on a rotator overnight at 4 °C in the presence of protease inhibitors. Following addition of protein-A/G agarose beads and washing, DNA was purified using the ChIP DNA Clean & Concentrator kit (Zymo Research) and used in qPCR or sequencing on a HiSeq4000 platform (Illumina). qPCRs were performed with the primers listed in Supplementary Data File 4. Where ChIP-seq was performed, at least 20 million reads were obtained, also for the relevant “input” samples. Raw sequencing reads (typically 50 nt-long) were analyzed using the HiChIP pipeline^78^, and peaks were called using MACS2^79^. Thresholded CHD6 ChIP-seq peaks (q-value <0.05) per each cell type and genotype are listed in Supplementary Data File 6. For plotting ChIP-seq signal coverage over select regions, *ngs.plot* was used^80^.

### Immunostaining and imaging

Cells were grown on coverslips, fixed in 4% PFA/PBS for 10 min at room temperature, washed in 1× PBS, permeabilized in 0.5% Triton-X/PBS for 5 min at room temperature, blocked in 1% BSA/PBS for 1 h before incubating with the primary antibody of choice for 2 h to overnight. Cells were next washed twice in 1x PBS for 5 min, before incubating with the appropriate secondary antisera for 1 h at room temperature. Nuclei were counterstained with DAPI (Sigma-Aldrich) for 5 min, washed, and coverslips mounted onto slides in Prolong Gold Antifade (Invitrogen). For image acquisition, a widefield Leica DMI 6000B with a HCX PL APO 63×/1.40 (Oil) objective was used, making sure exposure times were maintained constant across samples in each imaging session for the same immunostaining. Finally, images were analyzed using the Fiji suite^81^ as follows. First, background signal levels were subtracted using the embedded function (rolling ball function of 50-px radius with a sliding paraboloid and disabled smoothing), and the DAPI channel was used to determine the area of interest where signal would be quantified from. Measured mean signal intensities were used to generate plots in R or via InstantClue^82^. For bean plots, dots represent the mean of the dataset; for box plots, whiskers ends represent the top and bottom quantiles, respectively.

### Analysis of autophagy flux

To measure autophagy flux, iPSCs were transiently transfected with a plasmid coding for a tandemly-tagged GFP-LC3-mRFP^41^. 48 h after transfection, cells were incubated in starvation medium or complete FTDA medium for 2 h before being lifted using accutase diluted by F12 media and supplemented with 10 μM of the Y27632 inhibitor to prevent apoptosis. Next, iPSCs were pelleted and resuspended in 150 μl of complete FTDA or starvation medium again supplemented with Y27632 inhibitor to undergo FACS analysis. Cell were counted in a TC20 Automated cell counter (BioRad) and 100,000 cells of each genotype and treatment were stained with Zombie-NIR viability solution (1:1000; BioLegend) as per the manufacturer’s instructions to exclude dead cells from the analysis. iPSCs overexpressing GFP or RFP only were used in parallel as baseline controls, and non-transfected cells served as a negative control. Measurements were performed on a Cytek Aurora flow cytometer and data analysis was performed via the SpecroFlo software. The percentage of cells carrying fused autolysosomes was estimated as a proportion of mRFP-positive cells out of total number of mRFP/GFP-double positive cells from two experiments.

### Comet assays

Comet assays were performed according to a standard protocol^83^. Briefly, cells were treated with 30 µg/ml etoposide for 30 min, harvested with accutase to prepare a single-cell suspension, counted, and diluted to a cell density of ~2 × 10^4^ cells/ml in PBS without bivalent cations, on ice. Electrophoresis slides were covered with low-melting agarose, alkaline lysis was allowed to run overnight, before slides were submerged in rinse solution for 20 min, the solution exchanged another 2 times to ensure removal of salts and detergents, and finally submerged in an electrophoresis chamber filled with fresh wash buffer. Electrophoresis proceeded for 25 min at a constant current of 40 mA, before slides were neutralized in distilled water, placed in staining solution containing 2.5 µg/ml of propidium iodide for 20 min, and rinsed again in water. For analysis, *CometScore* [http://rexhoover.com/index.php?id=cometscore] was used with doublets or comets at slide edges discarded from analysis. The length and intensity of DNA “tails” relative to “heads” were used as proxies for the amount of DNA damage in individual nuclei.

### Generation of stable CHD6 overexpression lines, immunoprecipitation, and proteomics

Full-length *CHD6* cDNA lacking the stop codon was PCR-amplified from wild-type NCC cDNA and cloned into a piggyback backbone (Ka0717_pPb-hCMV-cHA-IRES Venus)^68^ between the *Mlu*I and *Spe*I restriction sites, thus positioning the *CHD6* cDNA in frame with a C-terminal HA-tag. The HSS-relevant mutation (C4800G) was introduced to the wt*CHD6* piggybac vector using site-directed mutagenesis. Wild-type iPSCs were transfected as described above, using the wt- or mut*CHD6*-containing piggybac together with a transposase-expressing vector enabling few random integrations of the construct into the genome. Individual clones were selected after clonal dilution and selection on the basis of high Venus signal. *CHD6* overexpression was confirmed by immunofluorescence and western blots using anti-HA antisera. Following overexpression, wt/mutCHD6 immunoprecipitation was performed on freshly-harvested doxycycline-induced iPSCs. First, cell nuclei were isolated by incubating cells for 15 min on ice in NIB buffer (15 mM Tris-HCL pH 7.5, 60 mM KCl, 15 mM NaCl, 5 mM MgCl_2_, 1 mM CaCl_2_, 250 mM sucrose) containing 0.3% NP-40. Nuclei were pelleted for 5 min 800 × *g* at 4 °C, washed twice in the same buffer, lysed for 10 min on ice in IP buffer (150 mM LiCl, 50 mM Tris-HCl pH 7.5, 1 mM EDTA, 0.5% Empigen) freshly supplemented with 2 mM sodium vanadate, 1× protease inhibitor cocktail (Roche), PMSF (10 µl), 0,5 mM DTT, 5 µl Caspase inhibitor III (Calbiochem), and 50 units Benzonase per ml of IP buffer, before preclearing cell debris by centrifugation at >15,000 × *g* at 4 °C. Finally, 1 mg of the lysate was incubated with anti-HA antisera overnight at 4 °C. Magnetic beads (Active Motif) were then washed once with 1× PBS-Tween and combined with the antibody-lysate mixture. Following a 2-h incubation at 4 °C, beads were separated on a magnetic rack and washed 5×, 5 min each in wash buffer (150 mM KCl, 5 mM MgCl_2_, 50 mM Tris-HCl pH 7.5, 0.5% NP-40) and another two times in wash buffer without NP-40. Captured proteins were predigested and eluted from the beads using digestion buffer (2 M Urea, 50 mM Tris-HCl pH 7.5, 1 mM DTT) supplemented with trypsin and eluted from the beads with elution buffer (2 M Urea, 50 mM Tris-HCl pH 7.5, 5 mM chloroacetamide) supplemented with trypsin and LysC, before subjected to mass-spectrometry on a Q-Exactive Plus Orbitrap platform coupled to an EASY nLC (Thermo Scientific). Peptides were loaded in solvent A (0.1% formic acid in water) onto an in-house packed analytical column (50 cm length, 75 µm I.D., filled with 2.7 µm Poroshell EC120 C1; Agilent); were chromatographically separated at a constant flow rate of 250 nL/min using the following gradient: 3–8% solvent B (0.1% formic acid in 80% acetonitrile) for 1 min, 8–30% solvent B for 39 min, 30–50% solvent B for 8 min, 50–95% solvent B for 0.3 min, followed by washing and column equilibration. The mass spectrometer was operated in data-dependent acquisition mode. An MS1 survey scan was acquired from 300–1750 *m/z* at a resolution of 70,000. The top 10 most abundant peptides were isolated within a 1.8 Th window and subjected to HCD fragmentation at a normalized collision energy of 27%. The AGC target was set to 5e^5^ charges, allowing a maximum injection time of 110 ms. Product ions were detected at a resolution of 35,000; Precursors were dynamically excluded for 10 s. All raw data were processed with Maxquant (v1.5.3.8) using default parameters. Briefly, MS2 spectra were searched against the Uniprot HUMAN.fasta (16.06.2017) database, including a list of common contaminants. False discovery rates on were estimated by a target-decoy approach to 1% (Protein FDR) and 1% (PSM FDR), respectively. The minimal peptide length was set to 7 amino acids and carbamidomethylation at cysteine residues was considered as a fixed modification. Oxidation (M) and Acetyl (Protein N-term) were included as variable modifications. The match-between runs option was enabled and LFQ quantification was enabled under default settings. The full list of peptide hits and their analysis is provided in Supplementary Data File 2.

### Western blotting

Western blotting was performed as previously described^45^. In brief, ~2 × 10^6^ cells were enzymatically detached or gently scraped off cell culture dishes, and pelleted for 5 min at 600 × *g*. The supernatant was discarded, and the pellet resuspended in 100 µl of ice-cold RIPA lysis buffer containing 1× protease inhibitor cocktail (Roche), incubated for 30 min on ice, and centrifuged for 15 min at >15,000 × *g* to pellet cell debris and collect supernatant. Total protein concentrations were determined using the Pierce BCA Protein Assay Kit (ThermoFisher Scientific), before extracts were stored at −80 °C. Proteins were resolved by SDS-PAGE, transferred onto membranes using the TransBlot Turbo setup (Bio-Rad), and detected using the antibodies and dilutions listed in Supplementary Table 1. Raw scans of the membranes from all western blots presented in this study can be found in the Source data file online.

### MNase nucleosome isolation and qPCR

iPSCs were grown in 6-well plates to ~80% confluency and rinsed with PBS prior to addition of 1 ml of freshly prepared permeabilization buffer (15 mM Tris/HCl pH 7.6; 60 mM KCl; 15 mM NaCl; 4 mM CaCl_2_; 0.5 mM EGTA; 300 mM sucrose; 0.2% NP-40; 0.5 mM β-mercaptoethanol supplemented with MNase (Sigma) at 1 μ/ml final concentration. MNase was added for 3 min at 37 °C, and stopped by addition of an equal volume of stop buffer (50 mM Tri/HCl pH 8.0; 20 mM EDTA; 1% SDS). Finally, 250 µg RNase A were added for 2 h at 37 °C, followed by addition of 250 µg proteinase K and incubation at 37 °C overnight. Next day, DNA was isolated via standard phenol-chloroform extraction, digestion efficiency was determined after electrophoresis in 1% agarose gels, and mononucleosomal DNA was isolated from the gel. Nucleosome occupancy was assessed by qPCR using the primers listed in Supplementary Data File 4.

### SA-ß-gal staining, organelle detection, and cell cycle analyses

Senescence-associated β-galactosidase assays (Cell Signaling) were performed as per the manufacturer’s instructions before manually counting positive cells. Acidic organelles were visualized using Lysotracker Deep Red (ThermoFisher) as per the manufacturer’s instructions. Cell cycle analysis was performed using live cell nuclear staining with RedDot1 (Biotum) as per the manufacturer’s recommendations, and flow cytometry data were analyzed using FlowJo [https://www.flowjo.com/]. For autophagy inhibition prior to etoposide treatment, 10 μM chloroquine or 2 μM spautin were used. In all cases, cells were pretreated with the inhibitor of choice for 2 h prior to etoposide treatment (30 μg/ml) for 30 min (10 μm chloroquine, 500 μM rapamycin or 250 nM bafilomycin A1). Cells were next rinsed with sterile PBS, and fresh medium with or without replenished inhibitors was added for overnight incubation. Finally, cells were collected using accutase and counted before performing comet assays.

### In vitro and in vivo migration assays

For in vitro migration (scratch) assays, wild-type and patient-derived NCCs were grown in 6-well plates and one scratch per well was manually inflicted using a sterile cell scraper. Cell migration into the scratch was monitored for up to 8 h in 2-h increments by brightfield microscopy. For in vivo migration assays, GFP-labeled wild-type and mutant iPSCs were differentiated into NCCs as described above. Using a blunt glass capillary, NCCs were lifted with the help of accutase and inserted into the developing anterior neural region (i.e., midbrain) of chicken embryos at stage HH9. Operated eggs were resealed with medical tape and incubated until stages HH20, when embryos were isolated and fixed in 4% PFA for immunofluorescence; GFP was detected using anti-GFP antisera (TP401, OriGene).

### In vivo CHD6 overexpression in chicken embryos

For in vivo CHD6 overexpression, electroporation of chicken embryos^37^ was performed on eggs from stage HH9-10 that were windowed and had the extraembryonic membrane partially removed. A vector containing mutant or wild type *CHD6* ORFs (Ka0717_pPb-hCMV-cHA-IRES Venus-CHD6m and Ka0717_pPb-hCMV-cHA-IRES Venus-CHD6m; this paper) were first mixed 9:1 with the plasmid encoding the transposition transactivator, and this mixture then mixed 2:1 with Fast Green solution (Sigma) and microinjected into the neural tubes of the embryos. The neural tube was then electroporated with 5x square pulses of 20 V/20 msec using the Intracel TSS20 Ovodyne electroporator, eggs were resealed with tape and re-incubated for 24 h, before tape was removed and 10 µl of 2 µg/ml doxycycline were added at the site of electroporation and eggs were sealed again and re-incubated until stage HH20, where heart dissections also took place. CHD6 overexpression was confirmed in transgenic embryos on the basis of GFP signal under an Olympus SZX16 stereomicroscope with an EXFO X-cite series 120PC Q apparatus. Under German law, experiments on fertilized eggs performed up until stage HH20 do not require animal ethics approval.

### CHD6 protein expression and purification and in vitro assays

Steps from cloning to protein expression and purification were carried out as described previously^48^. In brief, WT and mutant I1600M CHD6-HA cDNAs were cloned into pFastBac1 and the HA tag was replaced by a FLAG tag using PCR and Gibson assembly. SANT-SLIDE 2 domain constructs were generated by PCR with a primer introducing a FLAG tag at the 3′ end. Baculoviruses were all produced via the Bac-to-Bac Baculovirus Expression Systems manual (ThermoFisher). CHD6-FLAG protein constructs were purified from 4 liters of Sf9 cell cultures. Cells were resuspended in BC buffer (10% Glycerol, 20 mM HEPES, pH 7.9, 0.4 mM EDTA, freshly supplemented with β-mercaptoethanol and protease inhibitors) containing 250 mM NaCl (BC 250). Cells were lysed by 2 freeze–thaw cycles, and cell extracts were cleared by centrifugation prior to adding M2-affinity (anti-FLAG) gel (Sigma) for 4 h, at 4 °C. The extracts were then poured into Econo columns (Bio-Rad), and the flow-through was reapplied once. The retained M2-beads were washed twice with at least 10 resin volumes of: BC 250, BC 500, BC 1000 and once with about 10 resin volumes of BC 500, BC 250. The last wash was performed with 10 resin volumes of BC 100 prior to elution with BC 100 containing 0.25 mg/ml of FLAG-peptide (Sigma). Purified proteins were used in electrophoretic mobility shift assays (EMSA), in nucleosome mobilization/sliding, and in restriction enzyme accessibility (REA) assays using DNA and nucleosome substrates as described previously^48^, but using non-radioactive substrates. Detection and quantification of the substrates was achieved via staining with SYBR Gold Nucleic Acid Gel Stain (ThermoFisher).

### NanoDSF CHD6 thermal stability determination

For thermal unfolding experiments, full-length wildtype and I1600M CHD6 protein preparations were diluted to a final concentration of 150 nM in EX80 buffer, and 10 μL of each sample per capillary was prepared. The samples were loaded into high sensitivity UV capillaries, and measurements were carried out on a Prometheus NT.48 instrument with a temperature gradient set to increase 1 °C/min in the range of 20 °C to 90 °C and independently replicated twice.

### Assay for transposase-accessible chromatin using sequencing (ATAC-seq) and data analysis

Tn5 transposase-accessible chromatin was isolated from iPSCs according to the standard ATAC-seq protocol^84^ with one modification aiming at quantitative scaling of the resulting data. In brief, 10^5^ iPSCs per replicate we “spiked” with 200 *D. melanogaster* S2 cells, washed in 1× PBS and added to lysis buffer (10 mM Tris-HCl pH 7.4, 10 mM NaCl, 3 mM MgCl_2_, 0.1% NP-40, 0.1% Tween-20, and 0.01% digitonin) for 3 min^85^ to isolate nuclei. Nuclei were next washed in washing buffer (10 mM Tris-HCl pH 7.4, 10 mM NaCl, 3 mM MgCl_2_, 0.1% Tween-20) and pelleted by centrifugation. Isolated nuclei were resuspended in transposase reaction mix (25 μl 2× TD buffer, 16.5 μl 1× PBS, 0.5 μl 10% Tween-20, 0.5 μl 1% digitonin, 2.5 μl Tn5, and 5 μl nuclease-free H_2_O) and incubated at 37 °C for 30 min on Thermomixer under constant shaking at 1000 rpm. The transposition reaction was terminated by the addition of stop buffer (50 mM Tris-HCl pH 8, 10 mM EDTA, 1% SDS), and DNA purified using the DNA Clean & Concentrator kit (Zymo Research). Following standard library generation, samples were sequenced to >50 million read pairs on a NovaSeq6000 platform (Illumina). Read pairs were mapped to the hg38 and dm6 reference genome builds for human and Drosophila, respectively, using Bowtie2^86^. Non-primary, unmapped, duplicate, and mitochondrial reads were removed. Data mapping to the Drosophila genome were used in ChIPSeqSpike [https://bioconductor.org/packages/release/bioc/html/ChIPSeqSpike.html] for calculating scaling factors in order to produce RPKM-normalized scaled coverage.

### Statistics and reproducibility

*P*-values associated with Student’s/Welsch *t*-tests were calculated using GraphPad [http://graphpad.com/], and those associated with the Wilcoxon-Mann-Whitney test using the EDISON-WMW [https://ccb-compute2.cs.uni-saarland.de/wtest/] online tool. Unless otherwise stated, only *P*-values <0.01 were deemed as significant. Also note that for immunofluorescence analyses, representative images from one of at least two independent and converging experiments are displayed and quantified.

### Reporting summary

Further information on research design is available in the Nature Research Reporting Summary linked to this article.

## Supplementary information

Supplementary Information

Peer Review File

Reporting Summary

Description of Additional Supplementary Files

Supplementary Data 1

Supplementary Data 2

Supplementary Data 3

Supplementary Data 4

Supplementary Data 5

Supplementary Data 6

## Data Availability

All NGS data generated in this study are available at the NCBI Gene Expression Omnibus (GEO) via GSE135832 and GSE136057 accession numbers. Publicly available SMARCB1-knockdown RNA-seq from hESCs can also be found at GEO under accession number GSE128351, SMARCA4/B1/C1 iPSC ChIP-seq data under accession number GSE124903, CHD1/2 and CHD7 hESCs ChIP-seq were generated by the ENCODE Consortium and can be found under accession numbers GSE31477 and GSE29611, respectively, H3K4me3 ChIP-seq under accession number GSE29611, and DNase I hypersensitivity and FAIRE-seq data from hESCs under accession numbers GSE32970 and GSE35239, respectively. All proteomics data are available via the PRIDE ProteomeXchange Consortium repository^87^ under the dataset identifier PXD024803. All other data are available from the corresponding authors upon reasonable request. Source data are provided with this paper.

## Source data

Source Data
