# Supplementary material for: Overarching control of autophagy and DNA damage response by CHD6 revealed by modeling a rare human pathology
Source: Nat Commun. 2021 May 21;12:3014. doi: 10.1038/s41467-021-23327-1 (PMC8140133; doi:10.1038/s41467-021-23327-1)
Supplement: Supplementary file 1 — Supplementary Information [file 41467_2021_23327_MOESM1_ESM.pdf]

SUPPLEMENTARY INFORMATION

Overarching control of autophagy and DNA damage response by CHD6 revealed by modeling a rare human pathology

Yulia Kargapolova[1,#,*], Rizwan Rehimi[1,2], Hülya Kayserili[3], Joanna Brühl[4], Konstantinos Sofiadis[5], Anne Zirkel[1], Spiros Palikyras[5], Athanasia Mizi[5], Yun Li[6], Gökhan Yigit[6], Alexander Hoischen[7], Stefan Frank[1,8,#], Nicole Russ[1,8], Jonathan Trautwein[4], Bregje van Bon[7], Christian Gilissen[7], Magdalena Laugsch[1,#], Eduardo Gade Gusmao[5], Natasa Josipovic[5], Janine Altmüller[9], Peter Nürnberg[9], Gernot Längst[10], Frank J. Kaiser[11], Erwan Watrin[12], Han Brunner[7], Alvaro Rada-Iglesias[1,2,13], Leo Kurian[1,8], Bernd Wollnik[6,14], Karim Bouazoune[4,*] & Argyris Papantonis[1,5,*]

[1] *Center for Molecular Medicine Cologne (CMMC), University of Cologne, 50931 Cologne, Germany*
[2] *Cluster of Excellence Cellular Stress Responses in Age-associated Disorders (CECAD), University of Cologne, 50931 Cologne, Germany*
[3] *Medical Genetics Department, Koç University School of Medicine, Istanbul 34010, Turkey*
[4] *Institute for Molecular Biology und Tumour Research (IMT), Philipps-University Marburg, 35043 Marburg, Germany*
[5] *Institute of Pathology, University Medical Center Göttingen, 37075 Göttingen, Germany*
[6] *Institute of Human Genetics, University Medical Center Göttingen, 37075 Göttingen, Germany*
[7] *Department of Human Genetics, Radboud University Medical Center, 6525 GA Nijmegen, The Netherlands*
[8] *Institute of Neurophysiology, University of Cologne, 50931 Cologne, Germany*
[9] *Cologne Center for Genomics, University of Cologne, 50931 Cologne, Germany*
[10] *Biochemistry Centre Regensburg (BRC), University of Regensburg, 93053 Regensburg, Germany*
[11] *Institute of Human Genetics, University Hospital Essen, 45147 Essen, Germany*
[12] *Research Institute of Genetics and Development, Faculté de Médecine, 35043 Rennes, France*
[13] *Institute of Biomedicine and Biotechnology of Cantabria (IBBTEC), University of Cantabria, 39011 Santander, Spain*
[14] *Cluster of Excellence Multiscale Bioimaging: from Molecular Machines to Networks of Excitable Cells (MBExC), University of Göttingen, 37075 Göttingen, Germany*

[#] *Present address: Y.K., Heart Center, University Hospital Cologne, 50937 Cologne, Germany; S.F., Bayer AG, 42096 Wuppertal, Germany*; M.L., *Institute of Human Genetics, University of Heidelberg, 69120 Heidelberg, Germany*

*Correspondence to: AP: argyris.papantonis@med.uni-goettingen.de; YK: ykargapo@uni-koeln.de; KB: bouazoune@imt.uni-marburg.de

Contents

—This online supplement contains **Supplementary Figures 1-6** and **Supplementary Table 1**.

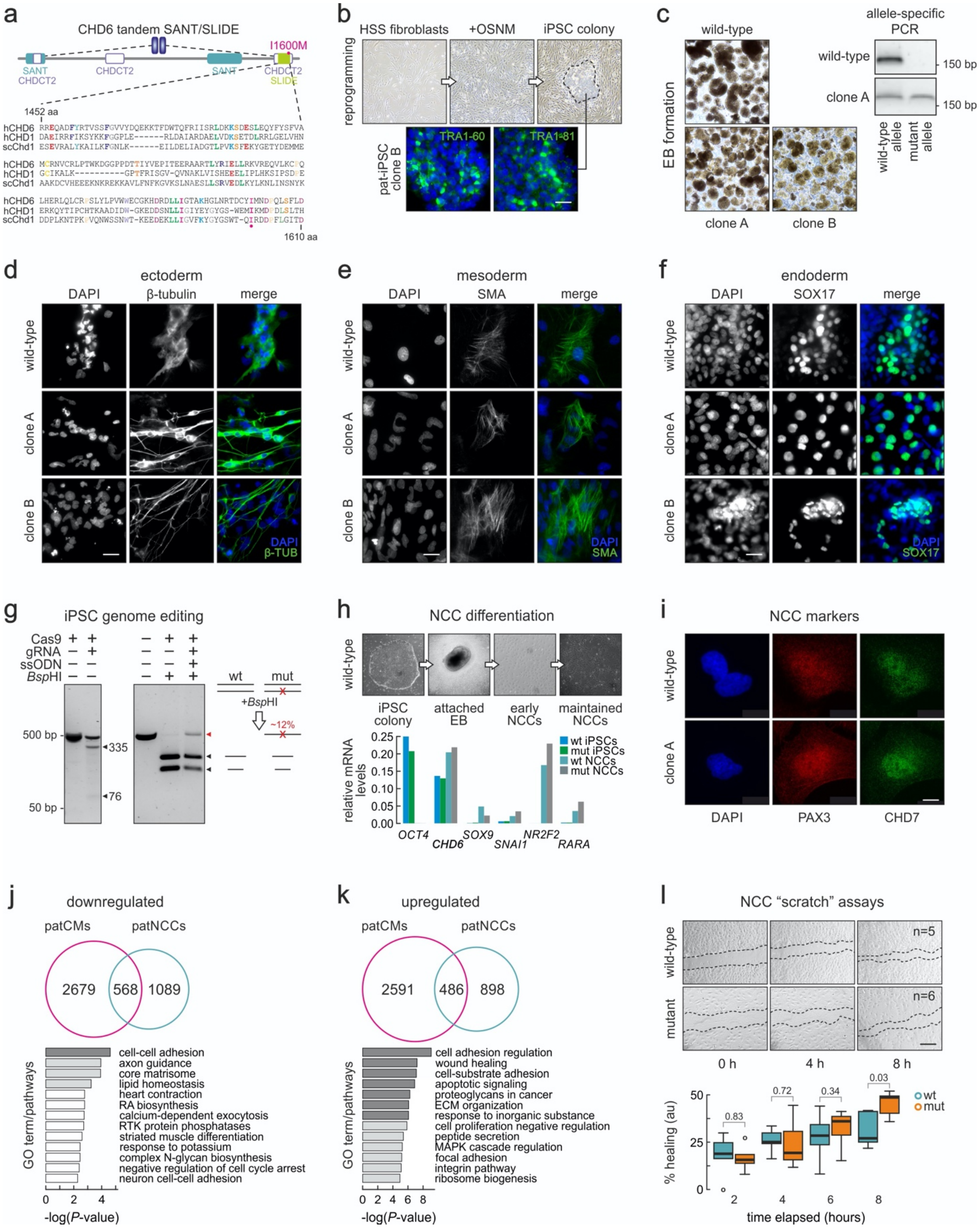
a
CHD6 tandem SANT/SLIDE
I1600M
SANT
CHDCT2
CHDCT2
SANT
CHDCT2
SLIDE
1452 aa
hCHD6
hCHD1
scChd1
1610 aa
b
HSS fibroblasts
+OSNM
iPSC colony
reprogramming
pat-iPSC clone B
TRA1-60
TRA1-81
c
wild-type
allele-specific PCR
wild-type
clone A
150 bp
150 bp
EB formation
clone A
clone B
wild-type allele
mutant allele
d
ectoderm
DAPI
β-tubulin
merge
wild-type
clone A
clone B
DAPI
β-TUB
e
mesoderm
DAPI
SMA
merge
DAPI
SMA
f
endoderm
DAPI
SOX17
merge
DAPI
SOX17
g
iPSC genome editing
Cas9
gRNA
ssODN
BspHI
wt
mut
+BspHI
~12%
500 bp
335
76
50 bp
h
NCC differentiation
iPSC colony
attached EB
early NCCs
maintained NCCs
relative mRNA levels
wt iPSCs
mut iPSCs
wt NCCs
mut NCCs
OCT4
CHD6
SOX9
SNAI1
NR2F2
RARA
i
NCC markers
DAPI
PAX3
CHD7
j
downregulated
patCMs
patNCCs
2679
568
1089
GO term/pathways
cell-cell adhesion
axon guidance
core matrisome
lipid homeostasis
heart contraction
RA biosynthesis
calcium-dependent exocytosis
RTK protein phosphatases
striated muscle differentiation
response to potassium
complex N-glycan biosynthesis
negative regulation of cell cycle arrest
neuron cell-cell adhesion
-log(P-value)
k
upregulated
patCMs
patNCCs
2591
486
898
cell adhesion regulation
wound healing
cell-substrate adhesion
apoptotic signaling
proteoglycans in cancer
ECM organization
response to inorganic substance
cell proliferation negative regulation
peptide secretion
MAPK cascade regulation
focal adhesion
integrin pathway
ribosome biogenesis
-log(P-value)
l
NCC "scratch" assays
wild-type
mutant
n=5
n=6
0 h
4 h
8 h
% healing (au)
wt
mut
0.83
0.72
0.34
0.03
time elapsed (hours)

Supplementary Fig. 1 Characterization of reprogrammed and genome-edited iPSC lines. a, SANT-related subdomains identified in CHD6 using the NCBI CDD with E-value=100 (*top*). The conservation of the I1600 CHD6 residue across CHD-family proteins is shown by alignment of the human (h) CHD6 and CHD1 with the *S. cerevisiae* (sc) Chd1 protein sequences (*below*). **b**, Representative brightfield images of patient-derived fibroblasts (*top left*) undergoing reprogramming via *OCT4*, *SOX2*, *NANOG*, and *MYC* overexpression (*top middle*), before single iPSC colonies are picked from the plate (dashed line; *top right*). Representative immunofluorescence images of patient-derived iPSC clones stained for the TRA1-60 (*bottom left*) and TRA1-81 markers (*bottom right*) in nuclei are counterstained with DAPI. Bar, 10 µm. **c**, Representative brightfield images of wild-type (*top*) and patient-derived iPSCs (*bottom*) giving rise to embryonic bodies (EBs). *Inset* (*top right*): allele-specific PCR confirmed expression of the mutant *CHD6* allele only in patient-derived cells. **d**, Representative immunofluorescence images of wild-type and two patient-derived iPSC clones (*middle* and *bottom rows*) differentiated into ectoderm and stained for β-tubulin. Bar, 10 µm. **e**, As in panel (d), but differentiated into mesoderm and stained for SMA. Bar, 10 µm. **f**, As in panel (d), but differentiated into endoderm and stained for SOX17. Bar, 10 µm. **g**, Agarose gel electrophoresis profiles of iPSC genomic DNA cut by Cas9 at the *CHD6* locus (*left*) and of wild-type and edited iPSCs diagnosed for carrying the HSS mutation via *Bsp*HI restriction digest. **h**, Representative brightfield images of iPSCs differentiated into NCCs (*top*) and RT-qPCR mean levels of lineage markers and *CHD6* (n=2 independent experiments; *bottom*). **i**, Representative immunofluorescence images of wild-type and mutant iPSC-derived NCCs stained for PAX6 (*left*) and CHD7 (*right*). Nuclei were counterstained with DAPI. Bar, 10 µm. **j**, Bar plot showing significantly enriched GO terms/pathways associated with downregulated genes (at least 0.6 $\log_2$-fold change, P_{adj}<0.05) shared by patient-derived CMs and NCCs. **k**, As in panel (j), but for upregulated genes shared by patient-derived CMs and NCCs. **l**, *In vitro* migration ("scratch") assays of wild-type (*top row*) and mutant NCCs (*bottom row*) and their quantification (boxplots show medians with whiskers indicating 95% confidence interval; *below*) over 8 h; unpaired two-tailed Student's t-test *P*-values are shown for each time point (the number, n, of independent assays performed is indicated). Bar, 50 µm.

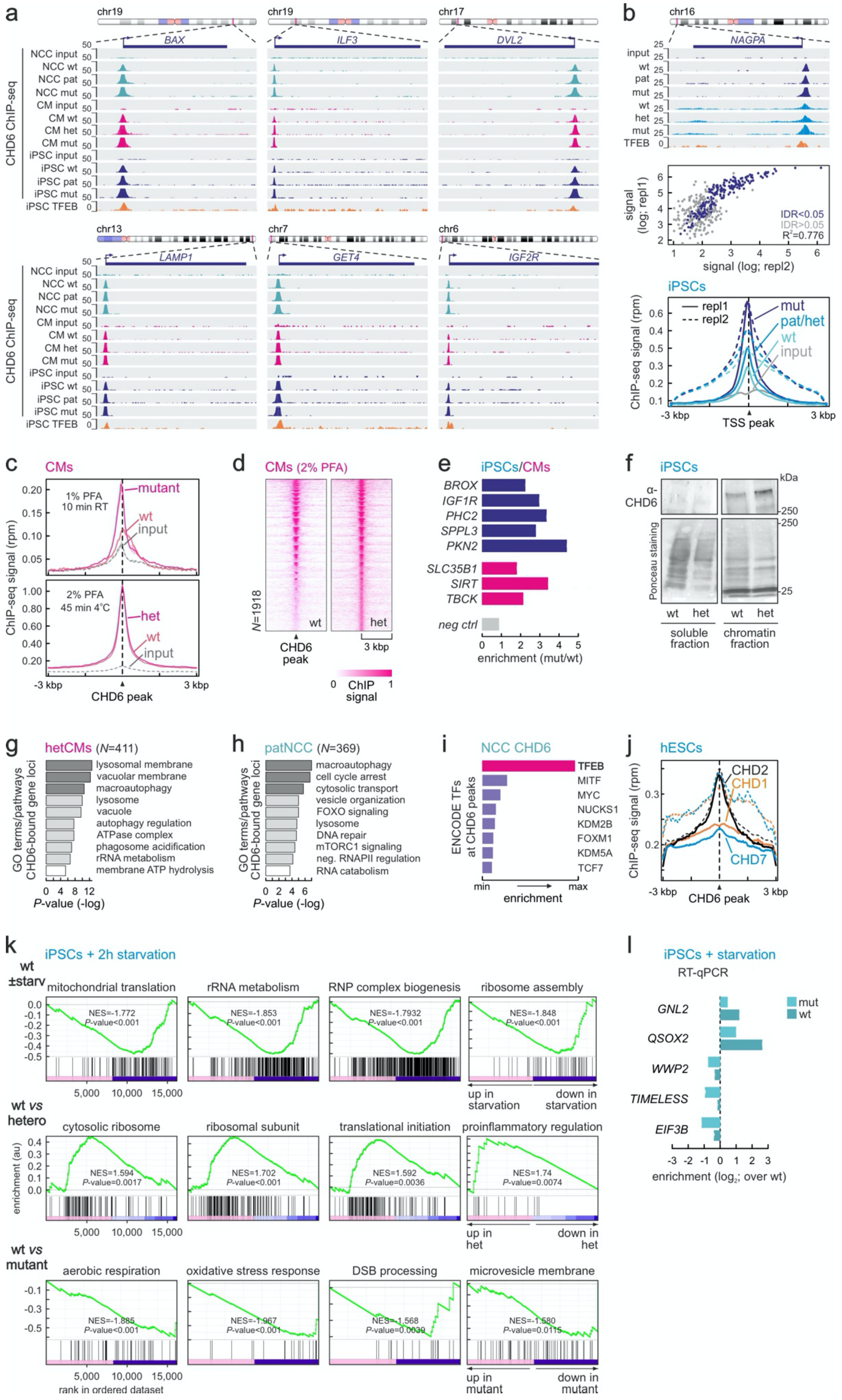
a
chr19
BAX
chr19
ILF3
chr17
DVL2
NCC input
NCC wt
NCC pat
NCC mut
CM input
CM wt
CM het
CM mut
iPSC input
iPSC wt
iPSC pat
iPSC mut
iPSC TFEB
CHD6 ChIP-seq
chr13
LAMP1
chr7
GET4
chr6
IGF2R
b
chr16
NAGPA
input
wt
pat
mut
wt
het
mut
TFEB
signal (log; repl1)
signal (log; repl2)
IDR<0.05
IDR>0.05
R²=0.776
iPSCs
repl1
repl2
mut
pat/het
wt
input
ChIP-seq signal (rpm)
-3 kbp
TSS peak
3 kbp
c
CMs
1% PFA 10 min RT
mutant
wt
input
2% PFA 45 min 4°C
het
wt
input
ChIP-seq signal (rpm)
-3 kbp
CHD6 peak
3 kbp
d
CMs (2% PFA)
N=1918
wt
het
CHD6 peak
3 kbp
ChIP signal
e
iPSCs/CMs
BROX
IGF1R
PHC2
SPPL3
PKN2
SLC35B1
SIRT
TBCK
neg ctrl
enrichment (mut/wt)
f
iPSCs
kDa
α-CHD6
Ponceau staining
-250
-250
-25
wt
het
wt
het
soluble fraction
chromatin fraction
g
hetCMs (N=411)
lysosomal membrane
vacuolar membrane
macroautophagy
lysosome
vacuole
autophagy regulation
ATPase complex
phagosome acidification
rRNA metabolism
membrane ATP hydrolysis
GO terms/pathways CHD6-bound gene loci
P-value (-log)
h
patNCC (N=369)
macroautophagy
cell cycle arrest
cytosolic transport
vesicle organization
FOXO signaling
lysosome
DNA repair
mTORC1 signaling
neg. RNAPII regulation
RNA catabolism
GO terms/pathways CHD6-bound gene loci
P-value (-log)
i
NCC CHD6
TFEB
MITF
MYC
NUCKS1
KDM2B
FOXM1
KDM5A
TCF7
ENCODE TFs at CHD6 peaks
min
max
enrichment
j
hESCs
CHD2
CHD1
CHD7
ChIP-seq signal (rpm)
-3 kbp
CHD6 peak
3 kbp
k
iPSCs + 2h starvation
wt ±starv
mitochondrial translation
NES=-1.772 P-value<0.001
rRNA metabolism
NES=-1.853 P-value<0.001
RNP complex biogenesis
NES=-1.7932 P-value<0.001
ribosome assembly
NES=-1.848 P-value<0.001
up in starvation
down in starvation
wt vs hetero
cytosolic ribosome
NES=1.594 P-value=0.0017
ribosomal subunit
NES=1.702 P-value<0.001
translational initiation
NES=1.592 P-value=0.0036
proinflammatory regulation
NES=1.74 P-value=0.0074
enrichment (au)
up in het
down in het
wt vs mutant
aerobic respiration
NES=-1.885 P-value<0.001
oxidative stress response
NES=-1.967 P-value<0.001
DSB processing
NES=-1.568 P-value=0.0039
microvesicle membrane
NES=-1.580 P-value=0.0115
rank in ordered dataset
up in mutant
down in mutant
l
iPSCs + starvation
RT-qPCR
GNL2
QSOX2
WWP2
TIMELESS
EIF3B
mut
wt
enrichment (log₂; over wt)

Supplementary Fig. 2 CHD6 ChIP-seq data analysis across the different cell types. a, Representative genome browser views of CHD6 ChIP-seq in wild-type (wt), heterozygous/ monoallelic mutant (het/mut) or patient-derived (pat) NCCs (*green*), CMs (*magenta*), and iPSCs (*blue*) aligned to iPSC TFEB ChIP-seq (*orange*). **b**, Representative genome browser views (*top*) of CHD6 ChIP-seq from two iPSC replicates (*dark/light blue*) in all genotypes aligned to TFEB ChIP-seq (*orange*). The scatter plot (*middle*) shows reproducibility of the two mutCHD6 replicates on the basis of IDR values; Spearmann's correlation coefficient (R^2) is also shown. The line plots (*bottom*) show mean ChIP-seq signal from wild-type (*light blue*) heterozygous/patient-derived (*blue*) and monoallelic-mutant iPSC replicates (*dark bluelue*) in the 6 kbp around 1770 CHD6-bound TSSs; input levels provide a control (*grey*). **c**, As in panel (b), but from wild-type (*light pink*) and monoallelic-/heterozygous-mutant CMs (*magenta*). CHD6 ChIP in the top panel was performed using 1% PFA for 10 min at room temperature, whereas in the bottom panel 2% PFA for 45 min at 4°C was used (yielding 271 and 642 wt peaks, respectively). **d**, Heatmaps showing ChIP-seq signal in the 6 kbp around CHD6-bound sites from wild-type (wt) or heterozygous mutant CMs (het) for all 1918 peaks detected. **e**, Bar plots showing ChIP-qPCR enrichment (mean, n=2 independent experiments) of mutant over wild-type CHD6 in iPSCs (*blue*) and CMs (*magenta*). **f**, Chromatin fractionation western blot showing CHD6 from equal numbers of wild-type and heterozygous mutant iPSCs localizing to chromatin. Ponceau stainings provide a loading control. **g**, Bar plots showing significantly-enriched GO terms/pathways for CHD6-bound genes in heterozygous mutant CMs. The number of genes analyzed (*N*) is given. **h**, As in panel (f), but for CHD6-bound genes in patient-derived NCCs. **i**, Bar plots showing signal enrichment for ENCODE transcription factor ChIP-seq data at CHD6-bound sites from mutant NCCs. **j**, Line plot showing CHD1 (*orange*), CHD2 (*black*), and CHD7 ChIP-seq signal (*blue*) in the 6 kbp around CHD6-bound sites from mutant iPSCs; signal distribution over all peaks in the respective dataset (e.g., CHD1 signal over all CHD1 peaks) provides a reference (*dashed lines*). **k**, Gene set enrichment analysis of ranked gene expression data from 3'-end RNA-seq data of serum-starved wild-type compared to serum-starved heterozygous (*middle row*) or monoallelic mutant iPSCs (*bottom row*); same analysis on wild-type iPSCs in the presence or absence of starvation provides a control (*top row*). **l**, Bar plots showing RT-qPCR mean expression changes (log_2) of exemplary genes in heterozygous (het) or monoallelic mutant iPSCs (mut) compared to wild-type (n=2 independent experiments) upon 2h-starvation.

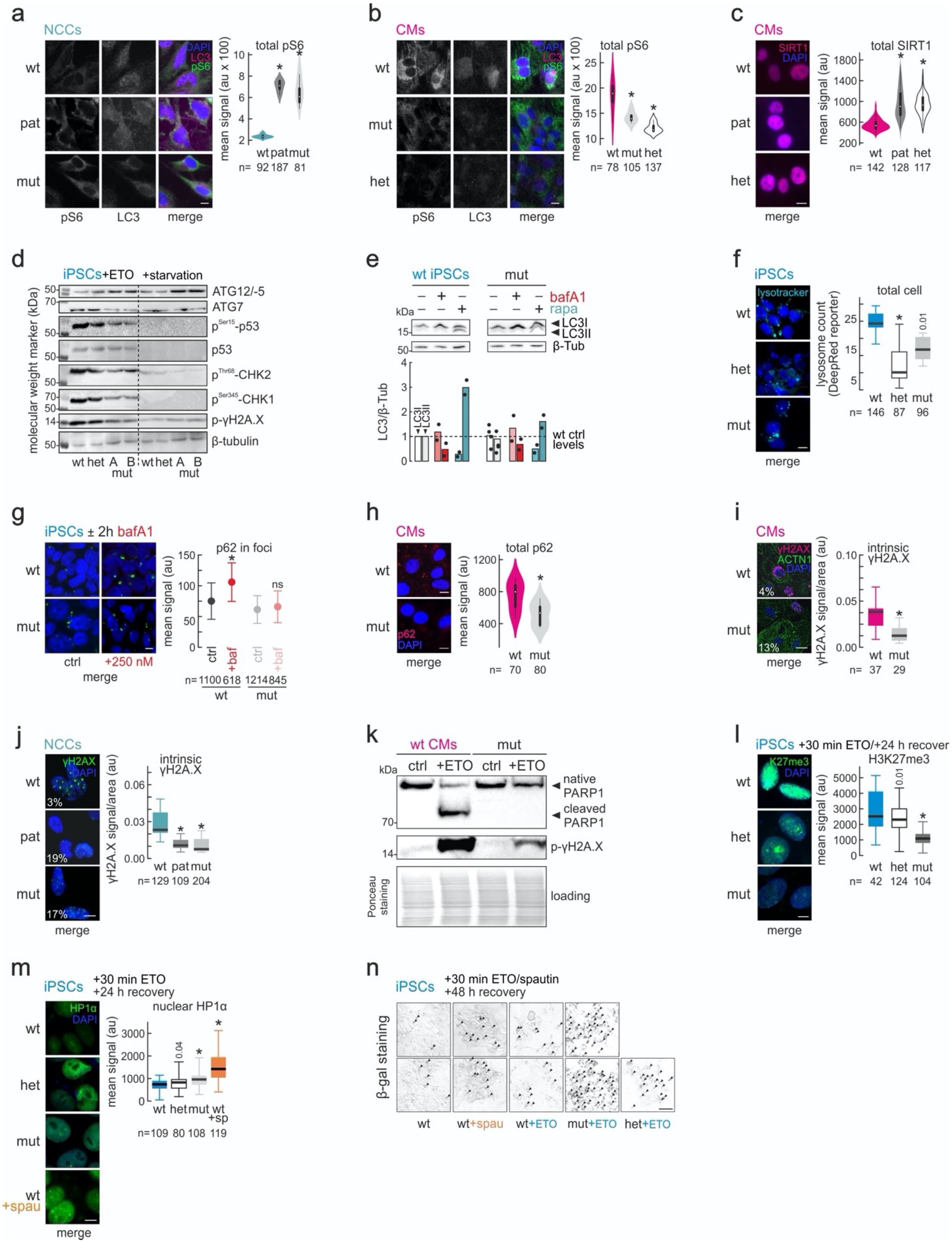

a
NCCs
wt
pat
mut
pS6
LC3
merge
DAPI
LC3
pS6
total pS6
mean signal (au x 100)
wt pat mut
n= 92 187 81
b
CMs
wt
mut
het
pS6
LC3
merge
total pS6
mean signal (au x 100)
wt mut het
n= 78 105 137
c
CMs
SIRT1
DAPI
wt
pat
het
merge
total SIRT1
mean signal (au)
wt pat het
n= 142 128 117
d
iPSCs+ETO
+starvation
molecular weight marker (kDa)
ATG12/-5
ATG7
pSer15-p53
p53
pThr68-CHK2
pSer345-CHK1
p-γH2A.X
β-tubulin
wt het A B wt het A B
mut
mut
e
wt iPSCs
mut
bafA1
rapa
kDa
LC3I
LC3II
β-Tub
LC3/β-Tub
wt ctrl levels
f
iPSCs
lysotracker
wt
het
mut
merge
total cell
lysosome count (DeepRed reporter)
wt het mut
n= 146 87 96
g
iPSCs ± 2h bafA1
wt
mut
ctrl
+250 nM
merge
p62 in foci
mean signal (au)
ctrl
+baf
ns
n= 1100 618 1214 845
wt
mut
h
CMs
wt
mut
p62
DAPI
merge
total p62
mean signal (au)
wt mut
n= 70 80
i
CMs
γH2AX
ACTN1
DAPI
wt
mut
4%
13%
merge
intrinsic γH2A.X
γH2A.X signal/area (au)
wt mut
n= 37 29
j
NCCs
γH2AX
DAPI
wt
pat
mut
3%
19%
17%
merge
intrinsic γH2A.X
γH2A.X signal/area (au)
wt pat mut
n= 129 109 204
k
wt CMs
mut
ctrl +ETO ctrl +ETO
kDa
native PARP1
cleaved PARP1
p-γH2A.X
Ponceau staining
loading
l
iPSCs +30 min ETO/+24 h recover
K27me3
DAPI
wt
het
mut
merge
H3K27me3
mean signal (au)
wt het mut
n= 42 124 104
m
iPSCs
+30 min ETO
+24 h recovery
HP1α
DAPI
wt
het
mut
wt +spau
merge
nuclear HP1α
mean signal (au)
wt het mut wt +sp
n=109 80 108 119
n
iPSCs
+30 min ETO/spautin
+48 h recovery
β-gal staining
wt
wt+spau
wt+ETO
mut+ETO
het+ETO

Supplementary Fig. 3 Autophagy, DNA damage response, and senescence induction in *CHD6*-mutant cells. **a,** Representative immunofluorescence images (*left*) of wild-type (wt), patient-derived (pat) or monoallelic mutant NCCs (mut) stained for LC3 and phospho-S6, and counterstained with DAPI from one experiment (but replicated independently). Bean plots (*right*) show median phospho-S6 levels (with thick lines indicating IQR, and thin lines indicating 95% confidence intervals). The number of cells analyzed (n) is inidicated below each plot. Bar, 5 µM. *: significantly different from wt; $P<0.01$, Wilcoxon-Mann-Whitney test. **b**, As in panel (a), but for total cell phospho-S6 levels in CMs. Bar, 5 µM. **c**, As in panel (a), but for total cell SIRT1 levels in CMs. Bar, 5 µM. **d**, Western blot analysis of proteins involved in autophagy and the DNA damage response in wild-type (wt), heterozygous (het) and two monoallelic-mutant iPSC lines (mutA,B) following etoposide treatment (*left*) or starvation (*right*) from at least 2 independent experiments; β-tubulin provides a loading control. **e**, Western blots (*top*) showing LC3I/III changes in wild-type and monoallelic mutant iPSCs treated with rapamycin (rapa) or bafilomycin (bafA1); β-tubulin provides a loading control. Normalized LC3I/II band intensities (mean, n=2 independent experiments) are plotted relative to wt levels (*bottom*). **f**, As in panel (a), but using the *in vivo* Lysotracker DeepRed reporter in iPSCs. Box plots show mean signal with whiskers indicating 95% confidence intervals. Bar, 5 µM. **g**, As in panel (f), but staining for p62 in wild-type or monoallelic mutant iPSCs in the presence or absence of bafilomycin A1 (bafA1). Plots show mean signal (±SD) in p62 puncta. *: significantly different mean to wt; $P<0.01$, unpaired two-tailed Welsch t-test. Bar, 5 µM. **h**, As in panel (a), but analyzing total cell p62 signal in CMs. **i**, As in panel (f), but staining for intrinsic γH2A.X in wild-type (wt) or monoallelic mutant CMs (mut). Bar, 5 µM. **j**, As in panel (i), but in wild-type (wt), patient-derived (pat) or monoallelic mutant NCCs (mut). Bar, 5 µM. **k**, Western blot of PARP1 and phosphorylated γH2A.X in wild-type (wt) or monoallelic mutant CMs (mut) with or without 30-min etoposide treatment. The positions of native and cleaved PARP1 are indicated (*arrowheads*); Ponceau staining provides a loading control. **l**, As in panel (f), but staining for H3K27me3 in iPSCs treated for 30 min with etoposide and allowed 48 h to recover. *significantly different mean to wt; $P<0.01$, unpaired two-tailed Welsch t-test. Bar, 5 µM. **m**, As in panel (l), but staining for HP1α, including autophagy inhibition using spautin (spau). Bar, 5 µM. **n**, Representative β-galactosidase stainings of wild-type (wt), monoallelic (mut) or heterozygous mutant iPSCs (het) treated or not with etoposide and/or spautin and allowed 24 h to recover; β-gal positive cells are indicated (*arrowheads*). Bar, 100 µM.

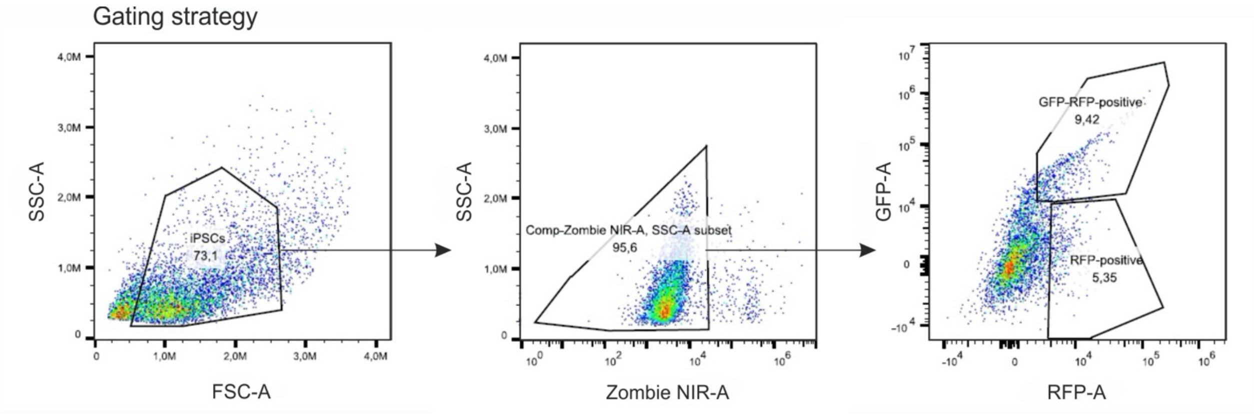

Supplementary Fig. 4 Gating strategy for assessing autophagy flux using cell sorting. Three consecutive steps for gating on the basis of light scattering (*left*), viability (*middle*), and GFP/RFP fluorescence levels (*right*). The numbers in each gate (*polygons*) indicate the percent of cells passing the threshold defined.

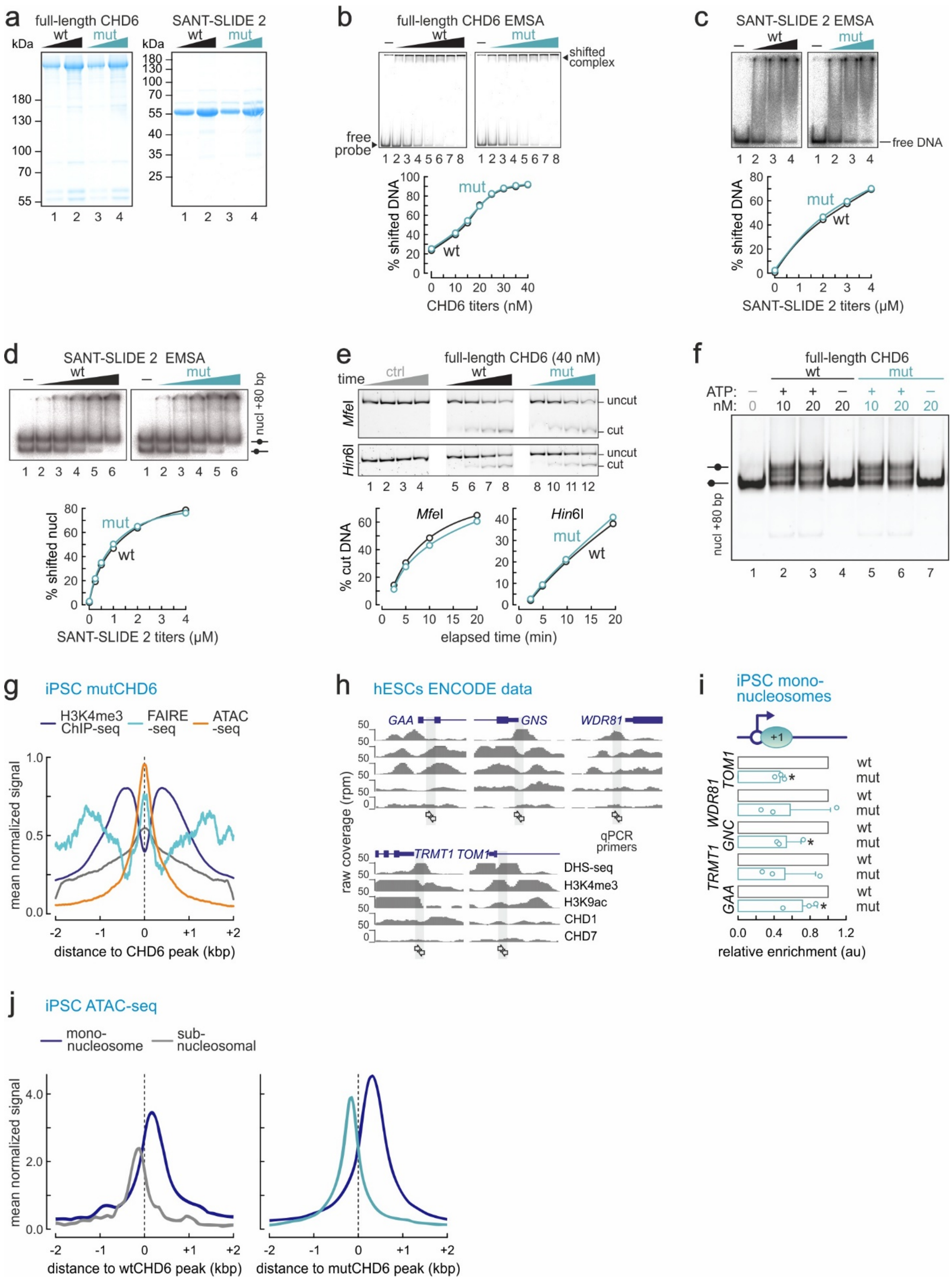

a
full-length CHD6
SANT-SLIDE 2
wt
mut
kDa
b
full-length CHD6 EMSA
shifted complex
free probe
% shifted DNA
CHD6 titers (nM)
c
SANT-SLIDE 2 EMSA
free DNA
SANT-SLIDE 2 titers (μM)
d
SANT-SLIDE 2 EMSA
nucl +80 bp
% shifted nucl
e
full-length CHD6 (40 nM)
time
ctrl
MfeI
Hin6I
uncut
cut
% cut DNA
elapsed time (min)
f
full-length CHD6
ATP:
nM:
g
iPSC mutCHD6
H3K4me3 ChIP-seq
FAIRE -seq
ATAC -seq
mean normalized signal
distance to CHD6 peak (kbp)
h
hESCs ENCODE data
GAA
GNS
WDR81
TRMT1
TOM1
raw coverage (rpm)
qPCR primers
DHS-seq
H3K4me3
H3K9ac
CHD1
CHD7
i
iPSC mono-nucleosomes
+1
relative enrichment (au)
GNC
j
iPSC ATAC-seq
mono-nucleosome
sub-nucleosomal
distance to wtCHD6 peak (kbp)
distance to mutCHD6 peak (kbp)

Supplementary Fig. 5 Effects of the I1600M CHD6 mutation on DNA binding and nucleosome remodeling. **a**, Coomassie-stained SDS-PAGE of purified recombinant wild-type (wt) and I1600M (mut) full-length CHD6 and its second putative SANT-SLIDE domain. The migration profiles of molecular weight markers (in kDa) are indicated. **b**, DNA EMSAs performed with increasing titers of wild-type (wt) or mutant (mut) full-length CHD6. Quantification of the assay is shown below. **c**, As in panel (b), but for the wild-type (wt) or mutant (mut) SANT-SLIDE 2 domain. **d**, As in panel (b), but with increasing titers of the wild-type (wt) or mutant(mut) CHD6 SANT-SLIDE 2 domain on a nucleosomal template. **e**, Restriction enzyme accessibility assays (REA; *top*) on a mononucleosome in the presence of 40 nm of wild-type (wt) or mutant full-length CHD6 (mut);Changes in DNA accessibility were monitored near the entry/exit site of the nucleosome with *MfeI* (+28-bp) or around the dyad axis with *Hin6I*, as indicated on the left; quantification is show below. **f**, Nucleosome sliding assays using an end-positioned nucleosome and increasing titers of wild-type (wt) or mutant full-length CHD6 (mut). **g**, Line plot showing mean distribution of H3K4me3 ChIP-seq (*blue*), FAIRE-seq (*green*), and ATAC-seq signal (*orange*) within the 4 kbp around mutCHD6 binding sites in iPSCs. **h**, Genome browser views showing hESC ENCODE DHS- and ChIP-seq data around five CHD6-bound gene promoters. Primer positions used in panel (i) are indicated by arrows. **i**, Bar plots showing mean relative MNase-qPCR signal enrichment (±SD; n=3 independent experiments) at CHD6-bound positions from panel (h) using mononucleosomes isolated from wild-type (wt) or monoallelic mutant (mut) iPSCs. *: significantly different from wt; $P<0.01$, two-tailed unpaired Student's t-test. **j**, Line plots showing mean ATAC-seq signal in the 4 kbp around wild-type (*left*) or mutant CHD6 ChIP-seq peaks (*right*). ATAC-seq reads corresponding to sub-nucleosomal DNA (<100 bp; *grey/green lines*) are plotted separately from those corresponding to nucleosomes (>150 bp; *blue lines*).

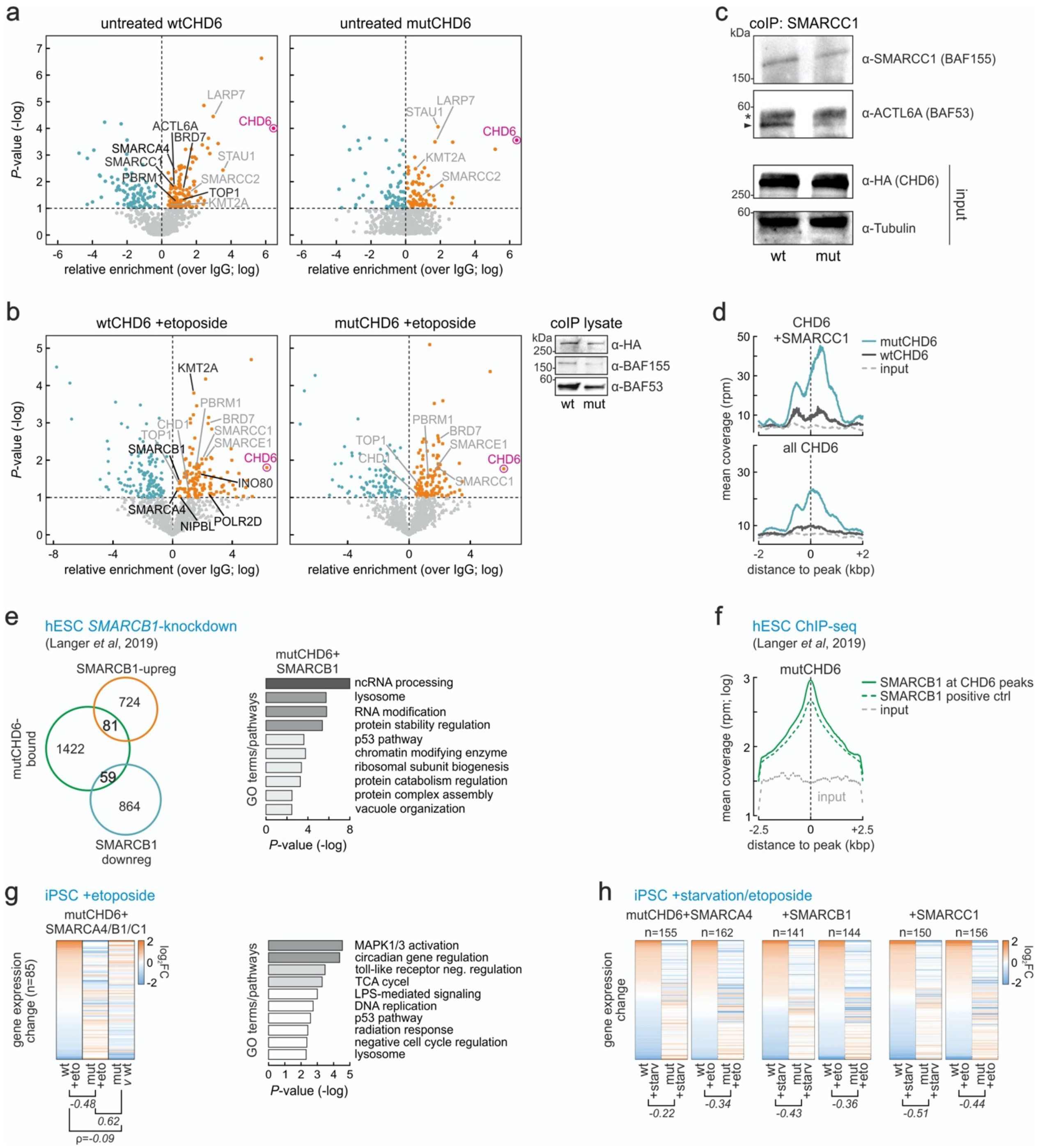
a
untreated wtCHD6
untreated mutCHD6
P-value (-log)
relative enrichment (over IgG; log)
LARP7
CHD6
ACTL6A
BRD7
SMARCA4
SMARCC1
PBRM1
STAU1
SMARCC2
TOP1
KMT2A
c
coIP: SMARCC1
kDa
α-SMARCC1 (BAF155)
α-ACTL6A (BAF53)
α-HA (CHD6)
α-Tubulin
input
wt
mut
b
wtCHD6 +etoposide
mutCHD6 +etoposide
coIP lysate
α-HA
α-BAF155
α-BAF53
SMARCE1
CHD1
SMARCB1
INO80
NIPBL
POLR2D
d
CHD6 +SMARCC1
all CHD6
mutCHD6
wtCHD6
input
mean coverage (rpm)
distance to peak (kbp)
e
hESC SMARCB1-knockdown
(Langer et al, 2019)
SMARCB1-upreg
mutCHD6-bound
SMARCB1 downreg
1422
724
81
59
864
mutCHD6+ SMARCB1
GO terms/pathways
ncRNA processing
lysosome
RNA modification
protein stability regulation
p53 pathway
chromatin modifying enzyme
ribosomal subunit biogenesis
protein catabolism regulation
protein complex assembly
vacuole organization
P-value (-log)
f
hESC ChIP-seq
(Langer et al, 2019)
mutCHD6
SMARCB1 at CHD6 peaks
SMARCB1 positive ctrl
input
mean coverage (rpm; log)
g
iPSC +etoposide
mutCHD6+ SMARCA4/B1/C1
gene expression change (n=85)
log2FC
wt +eto
mut +eto
mut v wt
-0.48
0.62
ρ=-0.09
MAPK1/3 activation
circadian gene regulation
toll-like receptor neg. regulation
TCA cycel
LPS-mediated signaling
DNA replication
p53 pathway
radiation response
negative cell cycle regulation
lysosome
h
iPSC +starvation/etoposide
mutCHD6+SMARCA4
+SMARCB1
+SMARCC1
n=155
n=162
n=141
n=144
n=150
n=156
gene expression change
wt +starv
mut +starv
wt +eto
mut +eto
-0.22
-0.34
-0.43
-0.36
-0.51
-0.44

Supplementary Fig. 6 Effects of the I1600M CHD6 mutation on co-factor recruitment and gene expression. **a**, Volcano plots of proteomics data following co-immunoprecipitation of proteins interacting with wild-type (*left*) or mutant CHD6 (*right*) obtained in iPSCs. Proteins significantly enriched compared to non-specific IgG controls are shown in orange, and chromatin remodeling subunits lost in the mutCHD6 interactome are highlighted (*black*) over shared interactors (*light grey*); enrichment of CHD6 in the data (*magenta*) provides a positive control. **b**, As in panel (a), but in iPSCs treated with etoposide for 2h. Western blots on the coIP input (*right*) show levels of wt/mutant CHD6 (anti-HA), SMARCC1 (BAF155) and ACTL6A (BAF53). **c**, Western blot of SMARCC1 co-immunoprecipitated by ACTL6A in iPSCs overexpressing wild-type (wt) or mutant CHD6 (mut); anti-HA and anti-tubulin blots provide loading controls. **d**, Line plots showing mean distribution of mutant (*light green*) and wild-type CHD6 ChIP-seq signal (*grey*) in the 4 kbp around shared CHD6/SMARCC1 (*top*) or all CHD6 peaks in iPSCs (*bottom*). Input signal (*dashed line*) provides a baseline. **e**, Venn diagrams (*left*) showing the overlap between CHD6-bound genes and genes differentially-regulated upon *SMARCB1(INI1)*-knockdown in hESCs. Bar plots (*right*) show significantly-enriched GO terms associated with CHD6-bound up-/downregulated genes. **f**, Line plot showing mean distribution of SMARCB1 ChIP-seq signal (*green*) in the 5 kbp around mutCHD6 binding sites in iPSCs. Signal distribution around all SMARCB1 peaks (*green dashed line*) provides a positive control, and input signal (*grey dashed line*) provides a baseline. **g,** Heatmaps showing changes in mRNA levels (log_2) of genes co-bound by mutCHD6 and SMARCA4/-B1/-C1 in response to 1-h etoposide treatment. Pearson's correlation coefficients for each pairwise comparison are shown (*below*). Bar plots (*right*) show significantly-enriched GO terms associated with these genes. **h**, As in panel (g), but for changes in the expression of genes co-bound by mutant CHD6 and SMARCA4 (*left*), -B1 (*middle*) or -C1 separately (*right*) in response to iPSC starvation or etoposide treatment.

Supplementary Table 1. Full list of antibodies and dilutions used.

Antibody	Dilution used	Provider	Cat. No.	Link to manufacturer's info page
**CHD6**	WB (1:2000), ChIP (5 µg/IP), IF (1:1000)	Bethyl	A301-221A	https://www.bethyl.com/product/A301-221A/CHD6+Antibody
**TFEB**	ChIP (5 µg/IP), IF (1:1000)	Bethyl	A303-673A	https://www.bethyl.com/product/A303-673A/TFEB+Antibody
**Phospho-ATM (Ser1981)**	WB (1:2000), IF (1:1000)	Cell Signaling	5883	https://www.cellsignal.com/products/primary-antibodies/phospho-atm-ser1981-d6h9-rabbit-mab/5883
**Phospho-ATR (Ser428)**	WB (1:2000), IF (1:1000)	Cell Signaling	2853	https://www.cellsignal.com/products/primary-antibodies/phospho-atr-ser428-antibody/2853
**Phospho-BRCA1 (Ser1524)**	WB (1:2000), IF (1:1000)	Cell Signaling	9009	https://www.cellsignal.com/products/primary-antibodies/phospho-brca1-ser1524-antibody/9009
**Phospho-Chk1 (Ser345)**	WB (1:2000), IF (1:1000)	Cell Signaling	2348	https://www.cellsignal.com/products/primary-antibodies/phospho-chk1-ser345-133d3-rabbit-mab/2348
**Phospho-Histone H2A.X (Ser139)**	WB (1:2000), IF (1:1000)	Cell Signaling	9718	https://www.cellsignal.com/products/primary-antibodies/phospho-histone-h2a-x-ser139-20e3-rabbit-mab/9718
**Phospho-Chk2 (Thr68)**	WB (1:2000), IF (1:1000)	Cell Signaling	2197	https://www.cellsignal.com/products/primary-antibodies/phospho-chk2-thr68-c13c1-rabbit-mab/2197
**Phospho-p53 (Ser15)**	WB (1:2000), IF (1:1000)	Cell Signaling	9286	https://www.cellsignal.com/products/primary-antibodies/phospho-p53-ser15-16g8-mouse-mab/9286
**αHP1**	IF (1:1000)	Active Motif	39977	https://www.activemotif.com/documents/tds/39977.pdf
**H3K27 me3**	IF (1:1000)	Active Motif	39155	https://www.activemotif.com/catalog/details/39155
**CHD6 (E-6)**	IF (1:1000)	Santa Cruz	sc-393445	https://www.scbt.com/p/chd6-antibody-e-6
**Anti-HA-tag**	IF (1:1000), IP (5 µg)	Thermo Fisher Scientific	26183	https://www.thermofisher.com/antibody/product/HA-Tag-Antibody-clone-2-2-2-14-Monoclonal/26183
**ß-Tubulin**	WB (1:2000)	Sigma-Aldrich	T0198	https://www.sigmaaldrich.com/catalog/product/sigma/t0198?lang=de&region=DE
**SMA**	IF (1:1000)	Abcam	ab14106	https://www.abcam.com/taglntransgelin-antibody-ab14106.html
**ATG12**	WB (1:2000)	Cell Signaling	4180	https://www.cellsignal.com/products/primary-antibodies/atg12-d88h11-rabbit-mab/4180
**ATG5**	WB (1:2000)	Cell Signaling	12994	https://www.cellsignal.com/products/primary-antibodies/atg5-d5f5u-rabbit-mab/12994
**ATG7**	WB (1:2000)	Cell Signaling	8558	https://www.cellsignal.com/products/primary-antibodies/atg7-d12b11-rabbit-mab/8558
**PARP1**	WB (1:2000)	Enzo	ALX-210-302	https://www.enzolifesciences.com/ALX-210-302/parp-1-polyclonal-antibody/

**LC3A/B**	WB (1:2000)	Cell Signaling	12741	https://www.cellsignal.com/products/primary-antibodies/lc3a-b-d3u4c-xp-rabbit-mab/12741
**NKX2.5**	IF (1:500)	R&D	AF2444	https://www.rndsystems.com/products/human-nkx25-antibody_af2444
**NANOG**	IF (1:500)	R&D	AF1997	https://www.rndsystems.com/products/human-nanog-antibody_af1997
**OCT4**	IF (1:500)	Santa Cruz	sc-5279	https://www.scbt.com/de/p/oct-3-4-antibody-c-10
**PAX3**	IF (1:500)	DSHB	PAX3-s	https://dshb.biology.uiowa.edu/PCRP-PAX3-1B3
**CHD7**	IF (1:500)	Abcam	ab31824	https://www.labome.com/product/Abcam/ab31824.html
**FOXD3**	IF (1:500)	Abcam	ab67758	https://www.abcam.com/foxd3-antibody-ab67758.html
**Phospho-S6 (Ser240/244)**	IF (1:500)	Cell Signaling	5364	https://www.cellsignal.com/products/primary-antibodies/phospho-s6-ribosomal-protein-ser240-244-d68f8-xp-rabbit-mab/5364
**MF20**	IF (1:500)	DSHB	MF20	https://dshb.biology.uiowa.edu/MF-20
**ß-Tubulin III (TUj1)**	IF (1:500)	Abcam	18207	https://www.abcam.com/beta-iii-tubulin-antibody-neuronal-marker-ab18207.html
**p62 (SQSTM1)**	IF (1:1000)	Biolegend	814801	https://www.biolegend.com/en-us/products/purified-anti-p62-sqstm1-antibody-11503
