# Supplementary material for: Overarching control of autophagy and DNA damage response by CHD6 revealed by modeling a rare human pathology
Source: Nat Commun. 2021 May 21;12:3014. doi: 10.1038/s41467-021-23327-1 (PMC8140133; doi:10.1038/s41467-021-23327-1)
Supplement: Supplementary file 3 — Reporting Summary [file 41467_2021_23327_MOESM3_ESM.pdf]

nature research

Corresponding author(s): A. Papantonis, K. Bouzaoune, Y. Kargapolova

Last updated by author(s): Feb 19, 2021

Reporting Summary

Nature Research wishes to improve the reproducibility of the work that we publish. This form provides structure for consistency and transparency in reporting. For further information on Nature Research policies, see Authors & Referees and the Editorial Policy Checklist.

Statistics

For all statistical analyses, confirm that the following items are present in the figure legend, table legend, main text, or Methods section.

n/a	Confirmed	
☐	☒	The exact sample size (*n*) for each experimental group/condition, given as a discrete number and unit of measurement
☐	☒	A statement on whether measurements were taken from distinct samples or whether the same sample was measured repeatedly
☐	☒	The statistical test(s) used AND whether they are one- or two-sided<br>*Only common tests should be described solely by name; describe more complex techniques in the Methods section.*
☒	☐	A description of all covariates tested
☐	☒	A description of any assumptions or corrections, such as tests of normality and adjustment for multiple comparisons
☐	☒	A full description of the statistical parameters including central tendency (e.g. means) or other basic estimates (e.g. regression coefficient) AND variation (e.g. standard deviation) or associated estimates of uncertainty (e.g. confidence intervals)
☐	☒	For null hypothesis testing, the test statistic (e.g. *F*, *t*, *r*) with confidence intervals, effect sizes, degrees of freedom and *P* value noted<br>*Give P values as exact values whenever suitable.*
☒	☐	For Bayesian analysis, information on the choice of priors and Markov chain Monte Carlo settings
☒	☐	For hierarchical and complex designs, identification of the appropriate level for tests and full reporting of outcomes
☒	☐	Estimates of effect sizes (e.g. Cohen's *d*, Pearson's *r*), indicating how they were calculated

Our web collection on statistics for biologists contains articles on many of the points above.

Software and code

Policy information about availability of computer code

Data collection	No software was used for data collection.
Data analysis	All analyses was performed using existing and validated bioinformatic tools. Details are given for each in the respective Methods section. Please note the following versions of software used: VarBank v2.6, ChAS suite v4.0, Kallisto v0.44.0, R v3.3.3, Bioconductor v3.4, the current online version of Metascape (www.metascape.org), STAR aligner v2.7.7a, HTSeq v0.11.1, DESeq2 v3.12, ngs.plot v2.63, INstantClue v0.9.240, SpecroFlo software v1.0, CometScore v2.0, Bowtie2 v24.2, and ChIPSeqkSpike v3.12

For manuscripts utilizing custom algorithms or software that are central to the research but not yet described in published literature, software must be made available to editors/reviewers. We strongly encourage code deposition in a community repository (e.g. GitHub). See the Nature Research guidelines for submitting code & software for further information.

Data

Policy information about availability of data

All manuscripts must include a data availability statement. This statement should provide the following information, where applicable:
- Accession codes, unique identifiers, or web links for publicly available datasets
- A list of figures that have associated raw data
- A description of any restrictions on data availability

All sequencing data generated in this study have been deposited in the NCBI Gene Expression Omnibus (GEO) repository under the accession numbers GSE135832 and GSE136057. This data deposition has now been made public.

Field-specific reporting

Please select the one below that is the best fit for your research. If you are not sure, read the appropriate sections before making your selection.

☒ Life sciences ☐ Behavioural & social sciences ☐ Ecological, evolutionary & environmental sciences

For a reference copy of the document with all sections, see nature.com/documents/nr-reporting-summary-flat.pdf

Life sciences study design

All studies must disclose on these points even when the disclosure is negative.

Sample size	For image quantification, typically sizes of approx. 100 cells were used per replica; for chicken embryos, >6 were used per condition. The former is aligned to what is typically used in immunofluorescence quantifications, while the latter also abides to an effort to reduce the usage of large chicken embryo numbers without compromising the statistical robustness of the experiments.
Data exclusions	No data exclusions were performed.
Replication	All experiments shown have been replicated at least in two independent replicas (typically three); replications were successful. For isogenic lines, we always use data from two independently-obtained single cell-derived clones, and present data representative of both.
Randomization	Given our exact phenotypes (e.g., all chicken used were essentially identical, and our iPSC lines predefined as wt or mutant) and predecided treatments, randomization was not applicable in our experiments.
Blinding	Blinding was only used in the chicken in vivo experiments, where R.R. was provided by Y.K. with pseudonamed plasmids for electroporation.

Reporting for specific materials, systems and methods

We require information from authors about some types of materials, experimental systems and methods used in many studies. Here, indicate whether each material, system or method listed is relevant to your study. If you are not sure if a list item applies to your research, read the appropriate section before selecting a response.

Materials & experimental systems

n/a	Involved in the study	
☐	☒	Antibodies
☐	☒	Eukaryotic cell lines
☒	☐	Palaeontology
☐	☒	Animals and other organisms
☐	☒	Human research participants
☒	☐	Clinical data

Methods

n/a	Involved in the study	
☐	☒	ChIP-seq
☐	☒	Flow cytometry
☒	☐	MRI-based neuroimaging

Antibodies

Antibodies used

CHD6 WB (1:2000), ChIP (5 µg/IP), IF (1:1000) Bethyl A301-221A
TFEB ChIP (5 µg/IP), IF (1:1000) Bethyl A303-673A
Phospho-ATM (Ser1981) WB (1:2000), IF (1:1000) Cell Signaling 5883
Phospho-ATR (Ser428) WB (1:2000), IF (1:1000) Cell Signaling 2853
Phospho-BRCA1 (Ser1524) WB (1:2000), IF (1:1000) Cell Signaling 9009
Phospho-Chk1 (Ser345) WB (1:2000), IF (1:1000) Cell Signaling 2348
Phospho-Histone H2A.X (Ser139) WB (1:2000), IF (1:1000) Cell Signaling 9718
Phospho-Chk2 (Thr68) WB (1:2000), IF (1:1000) Cell Signaling 2197
Phospho-p53 (Ser15) WB (1:2000), IF (1:1000) Cell Signaling 9286
αHP1 IF (1:1000) Active Motif 39977, 39978
H3K27Me3 IF (1:1000) Active Motif 39155, 39157
CHD6 (E-6) IF (1:1000) Santa Cruz sc-393445
HA-tag monoclonal antibody IF (1:1000), IP (5 µg) ThermoFisher Scientific 26183
ß-Tubulin WB (1:2000) Sigma-Aldrich T0198
SMA IF (1:1000) Abcam ab14106
ATG12 WB (1:2000) Cell Signaling 4180
ATG5 WB (1:2000) Cell Signaling 12994
ATG7 WB (1:2000) Cell Signaling 8558
PARP1 WB (1:2000) Enzo ALX-210-302
LC3 (A/B) WB (1:2000) Cell Signaling 12741
NKX2.5 IF (1:500) R&D AF2444
NANOG IF (1:500) R&D AF1997

	OCT4 IF (1:500) Santa Cruz sc-5279<br>PAX3 IF (1:500) DSHB PAX3-s<br>CHD7 IF (1:500) Abcam ab31824<br>FOXD3 IF (1:500) Abcam ab67758<br>S6-pS240/244 IF (1:500) Cell Signaling 5364<br>anti-MF20 IF (1:500) Hybridoma Bank, University of Iowa (supernatant)<br>ß-Tubulin III (TUj1) IF (1:500) Abcam 18207<br>anti p62 (SQSTM1) IF (1:1000) Biolegend 814801
Validation	Validation was on the basis of manufacturer-stated specificity and links to the relevant information are provided in Supplementary Data File 7.

Eukaryotic cell lines

Policy information about cell lines

Cell line source(s)	The iPSC line we used our basis for all our genome editing and in vitro differentiations is commercially available via the non-profit Coriell Repository (USA), and has been widely used in research (e.g., Liu et al, Nat Methods, 2015).
Authentication	iPSC authentication is provided by Coriell Repository upon purchase, but we also performed microarray karyotyping of all the clones used in this study (available in Supplementary Data File 1).
Mycoplasma contamination	Tested regularly (twice a year) as a requirement of our institute.
Commonly misidentified lines<br>(See ICLAC register)	Not applicable.

Animals and other organisms

Policy information about studies involving animals; ARRIVE guidelines recommended for reporting animal research

Laboratory animals	Chicken embryos, irrespective of sex, used until developmental stage HH20
Wild animals	No wild animals were used in this study.
Field-collected samples	No field-collected samples were used in this study.
Ethics oversight	Ethics approval for the experimental use of chicken embryos up to stage HH20 has been issued to Dr. Alvaro Rada-Iglesias (at the time in Center for Molecular Medicine (ZMMK), Univeristy of Cologne, Germany).

Note that full information on the approval of the study protocol must also be provided in the manuscript.

Human research participants

Policy information about studies involving human research participants

Population characteristics	The study initiated on the basis of genetic information (a missense CHD6 gene mutation) in a single patient of caucasian decent.
Recruitment	No recruitment strategy was implemented; the patient visited the clinic for diagnosis.
Ethics oversight	Ethics oversight for the consent, as well as the collection, storage, and usage of the patient biopsy is via the Ethics Borads of the Medical Faculties of the Universities of Cologne and Goettingen (Germany).

Note that full information on the approval of the study protocol must also be provided in the manuscript.

ChIP-seq

Data deposition

☒ Confirm that both raw and final processed data have been deposited in a public database such as GEO.

☒ Confirm that you have deposited or provided access to graph files (e.g. BED files) for the called peaks.

Data access links<br>*May remain private before publication.*	https://www.ncbi.nlm.nih.gov/geo/query/acc.cgi?acc=GSE135832<br>https://www.ncbi.nlm.nih.gov/geo/query/acc.cgi?acc=GSE135832
Files in database submission	We have deposited .fastq and .BED files in GEO; we also provide detailed peak lists for CHD6/TFEB ChIP-seq as Suppl Data Files (these can be used in UCSC directly as .BED files).
Genome browser session<br>(e.g. UCSC)	Not available.

Methodology

Replicates	The CHD6 ChIP-Seq experiment was performed in 9 cell lines: x3 Neural crest lines (wt, heterozyg. mutant, m.a. mutant) x3 iPSC lines (wt, heterozyg. mutant, m.a. mutant); x2 cardiomyocytes (wt, m.a. mutant) with short crosslinking and x2 cardiomyocytes (wt, heterozyg. mutant) with long crosslinking; TFEB ChIP was performed in m.a. CHD6 mutants, iPSC line
Sequencing depth	Sample_name # of uniquely mapped reads # of reads with multiple hits # of unmapped reads # total reads<br>K002000310_107511_S1_L003_R1_001.fastq.gz 21708688 2738070 527121 24973879<br>K002000310_107513_S2_L003_R1_001.fastq.gz 23986165 2993441 603589 27583195<br>K002000310_107515_S3_L003_R1_001.fastq.gz 17650243 4770769 2022408 24443420<br>K002000310_107517_S4_L003_R1_001.fastq.gz 14454742 3455207 1610744 19520693<br>K002000200_82885_1.fq 71051756 8515207 2666642 82233605<br>K002000174_78861_1.fq 34774326 3769906 1098283 39642515<br>K002000174_78863_1.fq 35144113 3559387 2045652 40749152<br>K002000297_98787_S30_L005_R1_001.fastq 40149301 4783517 1110114 46042932<br>K002000174_78859_1.fq 19537499 3670922 3862899 27071320<br>K002000297_98784_S27_L005_R1_001.fastq 36735846 10500941 3320924 50557711
Antibodies	CHD6 ChIP (5 μg/IP), Bethyl A301-221A Lot# A301-221A-1<br>TFEB ChIP (5 μg/IP), Bethyl A303-673A
Peak calling parameters	mapping: bwa-0.7.12//bwa aln -o 1 -l 32 -t 4 -k 2 -m 80000000 /projects/ag-papan/Yulia/annotation/references/allchr.fa /projects/ag-papan/Yulia/CHD6_ChIP_Rep1/K002000174_78861_1.fq.gz<br>peak calling: /projects/ag-papan/Yulia/Sources/chipseq_release/tools/macs2//bin//macs2 callpeak --bdg -t /projects/ag-papan/Yulia/CHD6_ChIP_Rep1/mapout/K002000174_78861_1.fq.gz.SE.U1.dedup.s1.bam -c /projects/ag-papan/Yulia/CHD6_ChIP_Rep1/mapout/K002000174_78859_1.fq.gz.SE.U1.dedup.s1.bam -n /projects/ag-papan/Yulia/CHD6_ChIP_Rep1/macs2out/K002000174_78861_1.fq.gz.SE_macs2 -f BAM -g hs -s 100 --keep-dup 1 -q 0.01
Data quality	Reads were quality checked with FastQC using standard criteria cuttoffs (typically >30).
Software	For data analysis we used HiChIP: A high-throughput pipeline for integrative analysis of ChIP-Seq data (bioinformaticstools.mayo.edu/research/hichipseq-pipeline/<br>Reads were quality checked with FastQC, mapped with BWA, low mapping quality reads and multimappers removed by Samtools, duplicates were removed by Picard, MACS2 was used as a peak finder.

Flow Cytometry

Plots

Confirm that:

☒ The axis labels state the marker and fluorochrome used (e.g. CD4-FITC).

☒ The axis scales are clearly visible. Include numbers along axes only for bottom left plot of group (a 'group' is an analysis of identical markers).

☒ All plots are contour plots with outliers or pseudocolor plots.

☒ A numerical value for number of cells or percentage (with statistics) is provided.

Methodology

Sample preparation	iPSCs were transiently transfected with a plasmid coding for a tandemly-tagged GFP-LC3-mRF; 48 h after transfection, cells were incubated in starvation medium or complete FTDA medium for 2 h before being lifted using accutase diluted by F12 media and supplemented with 10 μM of the Y27632 inhibitor to prevent apoptosis. Next, iPSCs were pelleted and resuspended in 150 μl of complete FTDA or starvation medium again supplemented with Y27632 inhibitor to undergo FACS analysis. Cell were counted in a TC20 Automated cell counter (BioRad) and 100,000 cells of each genotype and treatment were stained with Zombie-NIR viability solution (1:1000; BioLegend) to exclude dead cells from the analysis.
Instrument	Cytek Aurora flow cytometer.
Software	SpecroFlo software (Cytek proprietary).
Cell population abundance	iPSCs come from pure cell cultures, and were counted on a TC20 automatic cell counter before FACS.
Gating strategy	iPSCs were stained with Zombie-NIR viability solution (1:1000; BioLegend) to mark dead cells and exclude them from the analysis. iPSCs overexpressing GFP or RFP only were used in parallel as baseline controls for gating, and non-transfected cells served as a negative control. The percent of cells carrying fused autolysosomes was estimated as a proportion of mRFP-positive cells out of total number of mRFP/GFP-double positive cells from two experiments.

☒ Tick this box to confirm that a figure exemplifying the gating strategy is provided in the Supplementary Information.
