# Supplementary material for: Overarching control of autophagy and DNA damage response by CHD6 revealed by modeling a rare human pathology
Source: Nat Commun. 2021 May 21;12:3014. doi: 10.1038/s41467-021-23327-1 (PMC8140133; doi:10.1038/s41467-021-23327-1)
Supplement: Supplementary file 4 — Description of Additional Supplementary Files [file 41467_2021_23327_MOESM4_ESM.pdf]

Description of Additional Supplementary Files

File Name: Supplementary Data 1
Description: Summary of microarray-based karyotyping data of all iPSC lines used.

File Name: Supplementary Data 2
Description: List of peptide hits and statistical analysis of proteomics data from wild-type and mutant iPSCs untreated, treated with etoposide or subjected to starvation.

File Name: Supplementary Data 3
Description: Genes significantly up-/downregulated in 3' end RNA-seq data from wildtype, patient-derived or monoallelic mutant iPSCs after starvation or etoposide treatment.

File Name: Supplementary Data 4
Description: Sequences of oligonucleotides used as gRNAs, repair templates or PCR primers.

File Name: Supplementary Data 5
Description: Genes significantly up/downregulated in ribominus RNA-seq data from patient-derived or monoallelic mutant CMs or NCCs compared to wild-type counterparts.

File Name: Supplementary Data 6
Description: Lists of significant CHD6 ChIP-seq peaks and their genomic coordinates (hg19) from all cell types and genetic backgrounds.
