# Supplementary material for: Overarching control of autophagy and DNA damage response by CHD6 revealed by modeling a rare human pathology
Source: Nat Commun. 2021 May 21;12:3014. doi: 10.1038/s41467-021-23327-1 (PMC8140133; doi:10.1038/s41467-021-23327-1)
Supplement: Supplementary file 2 — Peer Review File [file 41467_2021_23327_MOESM2_ESM.docx]

**Editorial Note:** This manuscript has been previously reviewed at another journal that is not

operating a transparent peer review scheme. The manuscript was considered suitable for publication

without further review at *Nature Communications*.
